# kMoL: an open-source machine and federated learning library for drug discovery

**DOI:** 10.1186/s13321-025-00967-9

**Published:** 2025-02-25

**Authors:** Romeo Cozac, Haris Hasic, Jun Jin Choong, Vincent Richard, Loic Beheshti, Cyrille Froehlich, Takuto Koyama, Shigeyuki Matsumoto, Ryosuke Kojima, Hiroaki Iwata, Aki Hasegawa, Takao Otsuka, Yasushi Okuno

**Affiliations:** 1 Elix, Inc., 8–34 Yonbancho, Chiyoda-ku, Tokyo, 102–0081 Japan; 2 Graduate School of Medicine, Kyoto University, Shogoin-kawaharacho, Sakyo-ku, Kyoto, 606–8507 Japan

**Keywords:** Machine learning, Federated learning, Drug discovery, Deep learning, Graph convolutional networks, Distributed learning, Chemoinformatics

## Abstract

Machine learning is quickly becoming integral to drug discovery pipelines, particularly quantitative structure-activity relationship (QSAR) and absorption, distribution, metabolism, and excretion (ADME) tasks. Graph Convolutional Network (GCN) models have proven especially promising due to their inherent ability to model molecular structures using graph-based representations. However, maximizing the potential of such models in practice is challenging, as companies prioritize data privacy and security over collaboration initiatives to improve model performance and robustness. kMoL is an open-source machine learning library with integrated federated learning capabilities developed to address such challenges. Its key features include state-of-the-art model architectures, Bayesian optimization, explainability, and federated learning mechanisms. It demonstrates extensive customization possibilities, advanced security features, straightforward implementation of user-specific models, and high adaptability to custom datasets without additional programming requirements. kMoL is evaluated through locally trained benchmark settings and distributed federated learning experiments using various datasets to assess the features and flexibility of the library, as well as the ability to facilitate fast and practical experimentation. Additionally, results of these experiments provide further insights into the performance trade-offs associated with federated learning strategies, presenting valuable guidance for deploying machine learning models in a privacy-preserving manner within drug discovery pipelines. kMoL is available on GitHub at https://github.com/elix-tech/kmol.

**Scientific contribution** The primary scientific contribution of this research project is the introduction and evaluation of kMoL, an open-source machine learning library with integrated federated learning capabilities. By demonstrating advanced customization and security capabilities without additional programming requirements, kMoL represents an accessible yet secure open-source platform for collaborative drug discovery projects. Additionally, the experiment results provide further insights into the performance trade-offs associated with federated learning strategies, presenting valuable guidance for deploying machine learning models in a privacy-preserving manner within drug discovery pipelines.

## Introduction

Machine learning is quickly becoming an integral part of drug discovery pipelines. [1] It is utilized for various purposes, but it is particularly effective for quantitative structure-activity relationship (QSAR) [2, 3] and absorption, distribution, metabolism, and excretion (ADME) [4] tasks. Among different machine learning concepts utilized to address these tasks, Graph Convolutional Network (GCN) models have proven especially promising [5] due to their inherent ability to model molecular structures using graph-based representations, capturing intricate relationships between the atoms and bonds of the molecule. However, maximizing the potential of such models in practice is challenging, primarily because of data privacy and security concerns [6], which are significantly heightened when sharing data during collaborative efforts.

Data is considered a critical asset for any company, especially in the pharmaceutical industry, and its protection is paramount. On the other hand, collaboration between pharmaceutical companies, academic institutions, and research organizations has been, and continues to be, the driving force of innovation and progress. Therefore, data privacy and security concerns are constantly at odds with collaboration initiatives that aim to aggregate data from multiple sources to obtain more performant and robust machine learning models. This fundamental conflict of interest represents a significant barrier to possible scientific breakthroughs. Consequently, the potential of promising machine learning models like the GCN is harnessed only to a limited extent.

Several initiatives [7, 8] and libraries [9–13] have attempted to address data privacy and security concerns in collaborative projects using federated learning. As a part of the MELLODDY [7, 14] project, ten pharmaceutical companies realized aggregated improvements in machine learning model performance utilizing this strategy. The experiments were conducted using a combined dataset of approximately 2.6 billion confidential experimental activity data points, 21 million small molecules, and 40 thousand assays in on-target and secondary pharmacodynamics and pharmacokinetics. Similar experiments are also possible with NVIDIA Clara [8], a suite of computing platforms, software, and services for healthcare and life sciences. However, despite the impressive scale, these solutions have significant limitations, including requirements for consortium memberships, specific hardware, and substantial setup and customization. Other initiatives explore federated learning in drug discovery, however, in many cases, no codebase is provided, and when available, they primarily function as proofs of concept with limited features, flexibility and customization options, making them unsuitable for practical applications.

kMoL is an open-source machine learning library with integrated federated learning capabilities developed primarily for drug discovery pipelines. It demonstrates extensive customization options and advanced security features, allowing users to configure, optimize, and deploy custom machine learning models while securely sharing data, all without additional programming requirements. Since kMoL is open-source, it can be utilized instantaneously and irrespective of individual execution environments. Consequently, it is accessible to users from various backgrounds and adaptable to a variety of different datasets and use cases, allowing for fast and practical experimentation.

The primary scientific contribution of this research project is the introduction and evaluation of kMoL, which includes: (1) an explanation of the fundamental concepts of the library, providing a clear understanding of its simplified configuration interface, (2) a comprehensive evaluation using benchmarks and federated learning experiments across various datasets to assess its features and flexibility, and (3) insights into performance trade-offs associated with federated learning strategies, presenting valuable guidance on their effective use and limitations.

## Implementation

kMoL consists of a machine learning package and a federated learning package built on top of it. The underlying fundamental concepts of the library are:

- pipelines,
- data pre-processing, and
- data analysis and execution.

### Pipelines

Users interact with kMoL by running pipelines. The term pipeline is a comprehensive term that represents the lifecycle of a process within the library, from the time a user initiates a process up until they receive the response.

Pipelines are configured using JavaScript Object Notation (JSON) files. These files define parameters like runtime options, architecture details, and storage settings, among others. kMoL is developed to be easily extensible and customizable, allowing users to modify individual components without requiring a deep understanding of the entire library. Structuring configuration files using the JSON format significantly simplifies the customization process and serves as a superior storage medium for experiments compared to relying on command-line interface (CLI) arguments.

While pipelines can be customized to meet research requirements, most consist of two successive workflows: data pre-processing and data analysis and execution. The data pre-processing workflow consists of various data pre-processing actions and is automatically cached for performance reasons. (e.g., avoiding re-featurization of dataset samples in every epoch) The data analysis and execution workflow consists of training, inference, or validation actions performed on the pre-processed data.

### Data pre-processing

**Fig. 1:**
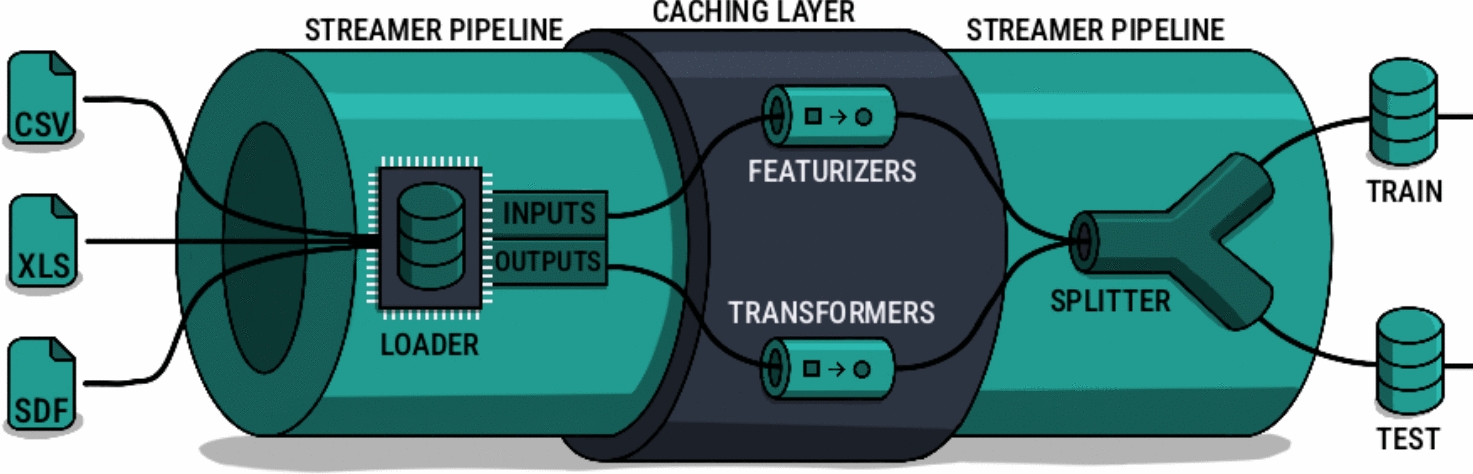
The data pre-processing workflow prepares raw data for analysis and execution. Users can mix and match five different components. Streamers manage how other lower-level components are interconnected. Loaders provide various ways to load data into memory from different file formats. Once in memory, featurizers and transformers process input and output features, respectively. Multiple featurizers and transformers can be chained together, and the final samples are cached to enhance performance across multiple epochs and experiments. Finally, splitters divide the processed samples into distinct groups

The primary objective of the data pre-processing workflow is to prepare raw dataset samples for the data analysis and execution workflow. As illustrated in Fig. 1, this workflow consists of five distinct components: loaders, featurizers, transformers, splitters, and streamers. All of these components can be customized and combined to meet requirements and be utilized in a variety of use cases:

1. Loaders load data from disk into memory:
  *CSV Loader* loads data from a comma-separated values (CSV) file.
  *Excel Loader* loads data from a Microsoft Excel spreadsheet (XLS) file.
  *SDF Loader* loads data from a structured data file (SDF).
2. Featurizers convert data to input features. Multiple featurizers can be applied successively, where the output of one featurizer serves as input for the next one.
  *Graph Featurizer* converts a molecular structure representation to a graph-based representation, where the atoms and bonds of the molecule correspond to the nodes and edges of the graph, respectively.
  *RDKit Descriptor Featurizer* converts a molecular structure representation to a vector of property descriptors computed with the RDKit [15] library.
  *Mordred Descriptor Featurizer* converts a molecular structure representation to a vector of property descriptors computed with the Mordred [16] descriptor calculator.
  *Circular Fingerprint Featurizer* [17] converts a molecular structure representation to a binary vector, where each component or bit of the output vector corresponds to the presence or absence of molecular substructures within a radius around each atom of the input molecule.
  *One-hot Encoder* converts a categorical data representation to a binary vector, where each category is represented by a vector of length equal to the number of unique categories with a single component or bit set to high or one and all other components or bits set to low or zero.
  *Tokenizer* performs a conversion similar to the *One-hot Encoder* applied to a DNA or amino acid sequence representation.
  *Bag of Words Featurizer* converts a DNA or amino acid sequence representation to a vector where each component represents the frequency of a token in the DNA or amino acid sequence.
  *Converter Featurizer* converts between different molecular structure representation formats. (e.g., InChI to SMILES)
  *Fixed Featurizer* divides an input representation by a constant value.
3. Transformers convert data to output features. Multiple transformers can be applied successively.
  *Log Normalizer* converts data points to their *log* values.
  *Min-max Normalizer* applies $min\text {-}max$ normalization to the data points.
  *Fixed Normalizer* divides the data points by a constant value.
  *Standardizer* applies $Z\text {-}score$ normalization to the data points.
  *Cut-off Transformer* converts continuous data points to binary values based on a threshold value.
  *One-hot Transformer* converts textual data points to numeric values.
4. Splitters divide data into distinct groups based on a criteria:
  *Index Splitter* divides the data into distinct groups based on their original order in the dataset without shuffling.
  *Random Splitter* divides the data into distinct groups randomly.
  *Stratified Splitter* divides the data into distinct groups randomly while maintaining the proportion of a specific output data value for each group.
  *Descriptor Splitter* performs the division similar to the *Stratified Splitter* utilizing descriptors computed with the RDKit [15] library.
  *Scaffold Balancer Splitter* utilizes the concept of molecular structure scaffolds to divide the data into distinct groups while maintaining an equal number of samples per molecular structure scaffold for each group.
  *Scaffold Divider Splitter* utilizes the concept of molecular structure scaffolds to divide the data into distinct groups that exclusively contain unique molecular structure scaffolds.
  *Butina Balancer Splitter* is a similarity-based splitter that utilizes the Butina clustering [18] approach to divide the data into distinct groups that contain similar molecular structures.
  *Butina Divider Splitter* is a similarity-based splitter that utilizes the Butina clustering [18] approach to divide the data into distinct groups that contain dissimilar molecular structures.
5. Streamers integrate loaders, featurizers, transformers, and splitters into a cohesive data pre-processing workflow:
  *Standard Streamer* is a general-purpose streamer that utilizes a data loader to iterate through individual dataset samples, featurize and transform them, and ultimately cache the results before splitting. Users can specify which data split should be used, what batch size should be used for collation, and whether to shuffle the data split.
  *Cross-validation Streamer* is a general-purpose streamer that performs similarly to the *Standard Streamer*, with the difference that it generates multiple cross-validation folds instead of a single one.

### Data analysis and execution

After the data pre-processing workflow, the pre-processed data is passed on to the data analysis and execution workflow. The operations of this workflow include training using fixed parameters, hyperparameter tuning using Bayesian optimization, standard validation, cross-validation, inference, and explanation [19, 20] of inference results. Federated learning operations of this workflow include launching a server and connecting to a server as a client.

#### Model architectures

kMoL supports a variety of machine learning model architectures adapted to different representations. Namely, it supports standard shallow neural network architectures, standard graph-based architectures for handling graph-featurized molecular structure data, and hybrid graph-based architectures that combine graph-featurized molecular structure data with vectorized data, such as molecular fingerprint [17] descriptors. Additionally, kMoL supports multi-modal architectures for use cases involving multiple inputs, such as combined training on ligand and protein sequence data.

##### Graph-based architectures

Most graph-based architectures are rooted in common fundamental concepts. First, the input molecular structure is converted to a graph-based representation. Next, the features of the resulting graph are processed through several graph convolutional layers, which aggregate information from neighboring graph nodes. Each graph layer applies changes to individual graph nodes, with deeper layers aggregating information from more distant nodes. Subsequently, a graph pooling layer is utilized to summarize and reduce the graph feature space. Graph features are ultimately flattened and passed through a shallow neural network. The kMoL implementation of graph-based architectures leverages PyTorch [21] and PyTorch Geometric [22], with the latter providing memory-efficient sparse implementations of numerous graph convolutional kernels. The following architectures are considered within the scope of this research:

- Graph Convolutional Network [23]
- Message Passing Network [24]
- Chebyshev Spectral Network [25]
- GraphSAGE Network [26]
- Weisfeiler-Leman Graph Network [27]
- Graph Isomorphism Network [28]
- ARMA Graph Network [29]
- Local Extremum Graph Network [30]
- GENeralized Graph Network [31]
- Cluster Graph Network [32]
- Feature-steered Graph Network [33]
- Graph Attention Network [34]
- Topology Adaptive Graph Network [35]
- Simple Graph Network [36]
- Triplet Message Passing Network [37]

##### Hybrid graph-based architectures

**Fig. 2:**
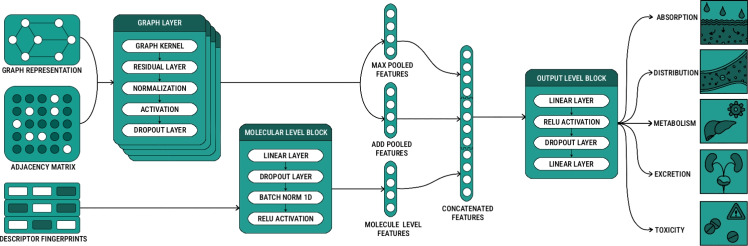
Overview of the hybrid graph-based architectures

Standard graph convolutional architectures focus exclusively on atom features and rely on the graph convolutional operator to derive global information about the molecule. In contrast, hybrid graph-based architectures pass molecular-level features directly as input in addition to the graph representation, as illustrated in Fig. 2.

First, the input molecular structure is converted to a graph representation. The resulting graph is then processed through several graph convolutional layers, which aggregate information from neighboring graph nodes. Edge features can be combined with atom features before passing through the graph operator in each layer. This is followed by a residual layer [38], a normalization layer with support for batch [39], layer [40], and graph [41] normalization, a configurable activation function, and a dropout [42] layer, where each layer serves as an input for the subsequent one. Ultimately, two feature sets are obtained. The first feature set is obtained by applying global maximum pooling, which takes the channel-wise maximum across the node dimension. The second feature set is obtained by applying global addition pooling, which sums node features across the node dimension.

In parallel, molecular-level features (e.g., RDKit [15], Mordred [16], or Circular Fingerprint [17] descriptors) are processed through a shallow neural network. The output of this network is then combined with the pooled graph features. Finally, the concatenated molecular and graph features are passed through a block consisting of two linear layers, a ReLU [43] activation function and a dropout [42] layer.

##### Multi-modal architectures

Multi-modal architectures allow for multiple data types within a single model. For example, if training is performed using a ChEMBL [44] assay with combined ligand and protein sequence data, a multi-modal architecture can accept both of these data types simultaneously within a single model. A graph-based or shallow neural network architecture can be configured for the ligand data, and classic one-dimensional convolutions or a shallow neural network utilizing bag-of-words features can be configured for the protein sequence data. The outputs are aggregated and ultimately passed through a shallow neural network.

#### Model training

Similar to machine learning model architectures, the training of models is easily customizable without additional programming requirements. The loss functions, learning rate schedulers, optimizers, and their respective parameters can be adjusted through JSON configuration files according to the relevant requirements. Moreover, the majority of the options that are available in PyTorch [21] and PyTorch Geometric [22] are also supported in kMoL, along with custom-developed components. (e.g., Evidential loss [45, 46] or AdaBelief optimizer [47]) Additionally, users can choose a subset from a list of 20 available metrics for model evaluation or logging purposes during training.

The relevant configurable options in kMoL can be tuned using Bayesian optimization. This tuning process encompasses architecture options like the model type, layer type, dropout rate, and normalization type, as well as training options like the optimizer type, criterion, and learning rate values. A Tree-structured Parzen Estimator (TPE) [48, 49] implemented using the Optuna [50] library is utilized for this purpose. During each optimization trial, the TPE fits one Gaussian Mixture Model (GMM) to the set of parameter values associated with the best objective values and another GMM to the remaining parameter values. Ultimately, the parameter value that maximizes the ratio between them is selected as optimal.

For inference, users have the option to enable visual explainability (XAI) tools implemented using IntegratedGradients [19] via Captum [20]. This feature creates images for input molecules where relevant atoms or bonds contributing to the predicted value are highlighted. Uncertainty estimation features like Monte Carlo Dropout [51] or Evidential Deep Learning [52, 53] are also supported.

#### Federated learning

During federated learning with kMoL, the communication between server and clients is facilitated using gRPC [54] and protocol buffers. Upon initialization, the server awaits the connection of multiple clients before commencing the training process. When available, the server dispatches a baseline model to each participant. The participating client then either fine-tunes the model received from the server or trains a new model using locally available data for a predetermined number of epochs. Upon completion of the training process, each participant sends the latest checkpoint back to the server for aggregation. After receiving the checkpoints from all clients, the server aggregates the checkpoints into a single global model. This process constitutes one round of federated learning, and the global model obtained at the end of each round serves as the baseline checkpoint for subsequent rounds.

The clients are continually sending a keep-alive signal to the server every minute during the federated learning training process. If a client becomes unreachable and the server fails to receive the signal from the client, it will halt the process. When a client authenticates, it receives a token that will be used for all subsequent operations. Unauthenticated machines (i.e., machines without a valid token) or dead clients (i.e., clients with an expired keep-alive signal) are not able to join the training process.

The communication is conducted utilizing the SSL/TLS protocol with server and client certificate chains. Insecure connections are also allowed for development purposes. In addition to direct communication between sites where checkpoints are stored locally, kMoL also supports cloud-based storage solutions such as Box [55] for managing checkpoint storage. Additionally, the server has the option to whitelist a set of trusted IP addresses that can join the process or blacklist a set of suspicious IP addresses that are prohibited from joining the process.

Differential privacy [56, 57] is supported in kMoL through the Opacus [58] library. It works by adding controlled random noise to model updates, reducing the likelihood that individual client data points can be inferred from the aggregated results. While this approach provides strong privacy guarantees, it may introduce trade-offs in model performance depending on the magnitude of the noise. This, among others, is outlined in a more comprehensive survey on privacy attacks in machine learning. [59]

## Results

The evaluation of kMoL is designed to showcase both the advantages and limitations of the library through practical application examples. Accordingly, the experiments are organized into two categories:

- local benchmarking experiments, and
- distributed federated learning experiments.

### Experiment design

Experiments are designed with a couple of objectives in mind. The first objective is to highlight the ability of kMoL to facilitate fast and practical experimentation, and consistently deliver well-performing machine learning models using a variety of datasets. The second objective is to demonstrate the strength of federated learning through the practical application of the library, providing valuable insights into when and how federated learning strategies can be effectively utilized and at what cost. By comparing the performance of these strategies to more traditional, local training strategies, the possible trade-offs are quantified and registered, and scenarios where federated learning is most advantageous are highlighted.

kMoL is evaluated using a variety of datasets. Architecture search and hyperparameter tuning are performed using Bayesian optimization, which employs a train-test split to evaluate candidate configurations and identify the optimal model setup. Subsequently, the optimal model is evaluated using five-fold cross-validation on the complete dataset to ensure robust performance. Within the scope of benchmarking experiments, kMoL is evaluated on MoleculeNet [5] datasets and compared to other state-of-the-art approaches. Within the scope of the federated learning experiments, kMoL is evaluated using Toxicity [60–63] and DruMAP [64] ADME datasets. Evaluation is performed by splitting the available data across multiple clients and training models following various federated learning strategies. The performance of these models is compared to the performance of baseline models trained locally and evaluated using cross-validation. Additional experiments utilizing the TDCommons [4] and ChEMBL [44] protein-ligand datasets are outlined in the supplementary information, sections S3 and S4, respectively.

### Benchmarking experiments

Benchmarking experiments were conducted using MoleculeNet [5] datasets for quantum mechanics, physical chemistry, biophysics, and physiology. Bayesian optimization was performed for architecture search and hyperparameter tuning, with 25 trials run for each dataset and 100 epochs per trial. Five-fold cross-validation was used to evaluate the best-performing models and ensure robust performance. Architecture and hyperparameter details for each model are outlined in the supplementary information, section S1. Extending upon previous work reported in GEM [65] and Uni-Mol [66], a scaffold split where scaffolds are unique to each fold was employed to ensure a consistent comparison between kMoL and existing benchmarks. Performance results are presented in Tables 1 and 2 for the classification and regression tasks, respectively. The $ROC\text {-}AUC$ metric is reported for classification tasks, and the $R^2$ metric for regression tasks. According to experiment results, machine learning models trained using kMoL are at least on par with the state-of-the-art, a quality prerequisite for federated learning experiments.

**Table 1:** Benchmark results on MoleculeNet [5] classification tasks

	SIDER	ClinTox	BACE	BBBP	Tox21	Toxcast	HIV	MUV	PCBA
Number of Samples	1,427	1,478	1,513	2,039	7,831	8,575	41,127	93,087	437,929
Number of Tasks	27	2	1	1	12	617	1	17	128
ROC-AUC (%)									
D-MPNN [67]	57.0	90.6	80.9	71.0	75.9	65.5	77.1	78.6	86.2
AttentiveFP [68]	60.6	84.7	78.4	64.3	76.1	63.7	75.7	76.6	80.1
N-GramRF [69]	66.8	77.5	77.9	69.7	74.3	–	77.2	76.9	–
N-GramXGB [69]	65.5	87.5	79.1	69.1	75.8	–	78.7	74.8	–
PretrainGNN [70]	62.7	72.6	84.5	68.7	78.1	65.7	79.9	81.3	86.0
GROVERbase [71]	64.8	81.2	82.6	70.0	74.3	65.4	62.5	67.3	76.5
GROVERlarge [71]	65.4	76.2	81.0	69.5	73.5	65.3	68.2	67.3	83.0
GraphMVP [72]	63.9	79.1	81.2	72.4	75.9	63.1	77.0	77.7	–
MolCLR [73]	58.9	91.2	82.4	72.2	75.0	–	78.1	79.6	–
GEM [65]	67.2	90.1	85.6	72.4	78.1	69.2	80.6	81.7	86.6
Mole-BERT [74]	62.8	77.0	80.8	67.6	76.8	64.3	78.2	78.6	–
Uni-Mol [66]	65.9	**91.9**	85.7	72.9	79.6	69.6	80.8	82.1	**88.5**
kMoL	**67.9** ± **3.3**	90.0 ± 3.6	**87.6** ± **2.2**	**90.5** ± **1.3**	**84.9** ± **2.67**	**77.13** ± **0.02**	**81.3** ± **1.5**	**83.2** ± **5.9**	83.7 ± 1.6

Bold values indicate the best-performing results for each dataset

**Table 2:** Benchmark results on MoleculeNet [5] regression tasks

	FreeSolv	ESOL	Lipophilicity	QM7	QM8
Number of Samples	642	1,128	4,200	6,830	21,786
Number of Tasks	1	1	1	1	12

	RMSE			MAE	
D-MPNN [67]	2.082	1.050	0.683	103.5	0.019
AttentiveFP [68]	2.073	0.877	0.721	72.0	0.018
N-GramRF [69]	2.688	1.074	0.812	92.8	0.024
N-GramXGB [69]	5.061	1.083	2.072	81.9	0.022
PretrainGNN [70]	2.764	1.100	0.739	113.2	0.020
GROVERbase [71]	2.176	0.983	0.817	94.5	0.022
GROVERlarge [71]	2.272	0.895	0.823	92.0	0.022
GraphMVP [72]	–	1.029	0.681	–	–
MolCLR [73]	2.594	1.271	0.691	66.8	0.018
GEM [65]	1.877	0.798	0.660	58.9	0.017
Mole-BERT [74]	–	1.015	0.676	–	–
Uni-Mol [66]	1.480	0.788	0.603	**41.8**	0.016
kMoL	**1.28 ± 0.09**	**0.70 ± 0.07**	**0.59 ± 0.04**	44.9 ± 5.2	**0.015 ± 0.002**

Bold values indicate the best-performing results for each dataset

### Federated learning experiments

Datasets used for federated learning experiments can be grouped into two distinct categories: Toxicity and ADME. The Toxicity category encompasses the Tox21 [60] and AMES [61–63] datasets, while the ADME category includes the Intrinsic Clearance (Human), Fraction Excreted Unchanged (Human), Brain Homogenate Binding (Mammal), Plasma Protein Binding (Human and Rat), P-gp Net Efflux Ratio (Human), Permeability (Human), Blood/Plasma Ratio (Rat), and Solubility datasets, all of which are a part of the DruMAP [64] analysis platform. The details of these datasets are presented in Table 3.

**Table 3:** Datasets used in federated learning experiments

Category	Name	Abbreviation	Number of Samples	Task
Toxicity	Tox21 [60]	Tox21	7,831	C
Toxicity	AMES [61–63]	AMES	7,441	C
ADME	Intrinsic Clearance (Human)	CLint	5,216	R
ADME	Fraction Excreted Unchanged (Human)	FeHuman	340	C
ADME	Brain Homogenate Binding (Mammal)	FuBrain	580	R
ADME	Plasma Protein Binding (Human)	FupHuman	2,559	R
ADME	Plasma Protein Binding (Rat)	FupRat	539	R
ADME	P-gp Net Efflux Ratio (Human)	NER-LLC	445	C
ADME	Papp Caco-2 Permeability (Human)	PappCaco2	4,294	C + R
ADME	Papp LLC-PK1 Permeability (Human)	Papp-LLC	461	R
ADME	Blood/Plasma Ratio (Rat)	RbRat	162	R
ADME	Solubility (pH 7.4)	Solubility	514	C

Tasks for federated learning experiments are denoted with C for classification, R for regression, and C + R for classification and regression

Bayesian optimization and cross-validation were performed similarly to the benchmarking experiments. However, in case of models trained on datasets with fewer samples (e.g., RbRat), cross-validation was repeated multiple times using different random seed values, and the average value is ultimately reported. Models trained on the Tox21, AMES, FupHuman, FuBrain, CLint, and PappCaco2 datasets were evaluated using one distinct five-fold cross-validation split. In contrast, models trained on the FupRat, Papp-LLC, NER-LLC, and FeHuman datasets were evaluated on five distinct five-fold cross-validation splits, while the model trained on the RbRat dataset was evaluated on ten distinct five-fold cross-validation splits. Cross-validation results are presented in Table 4. The $ROC\text {-}AUC$ metric is reported for classification tasks, and the $R^2$ metric for regression tasks. The metric values are formatted as $x \pm y$, where *x* denotes the mean value across all cross-validation folds, and *y* denotes the 95% confidence interval where $z=1.96$. These local training results serve as baseline metrics for distributed federated learning experiments.

**Table 4:** Cross-validation results for federated learning experiments

Dataset	Metric	Metric Value
Tox21	$ROC\text {-}AUC$	0.871 ± 0.013
AMES	$ROC\text {-}AUC$	0.893 ± 0.011
FeHuman	$ROC\text {-}AUC$	0.865 ± 0.039
NER-LLC	$ROC\text {-}AUC$	0.792 ± 0.041
PappCaco2	$ROC\text {-}AUC$	0.865 ± 0.005
Solubility	$ROC\text {-}AUC$	0.873 ± 0.022
CLint	$R^2$	0.458 ± 0.028
FuBrain	$R^2$	0.607 ± 0.077
FupHuman	$R^2$	0.663 ± 0.033
FupRat	$R^2$	0.602 ± 0.090
PappCaco2	$R^2$	0.505 ± 0.010
Papp-LLC	$R^2$	0.604 ± 0.080
RbRat	$R^2$	0.521 ± 0.149

**Fig. 3:**
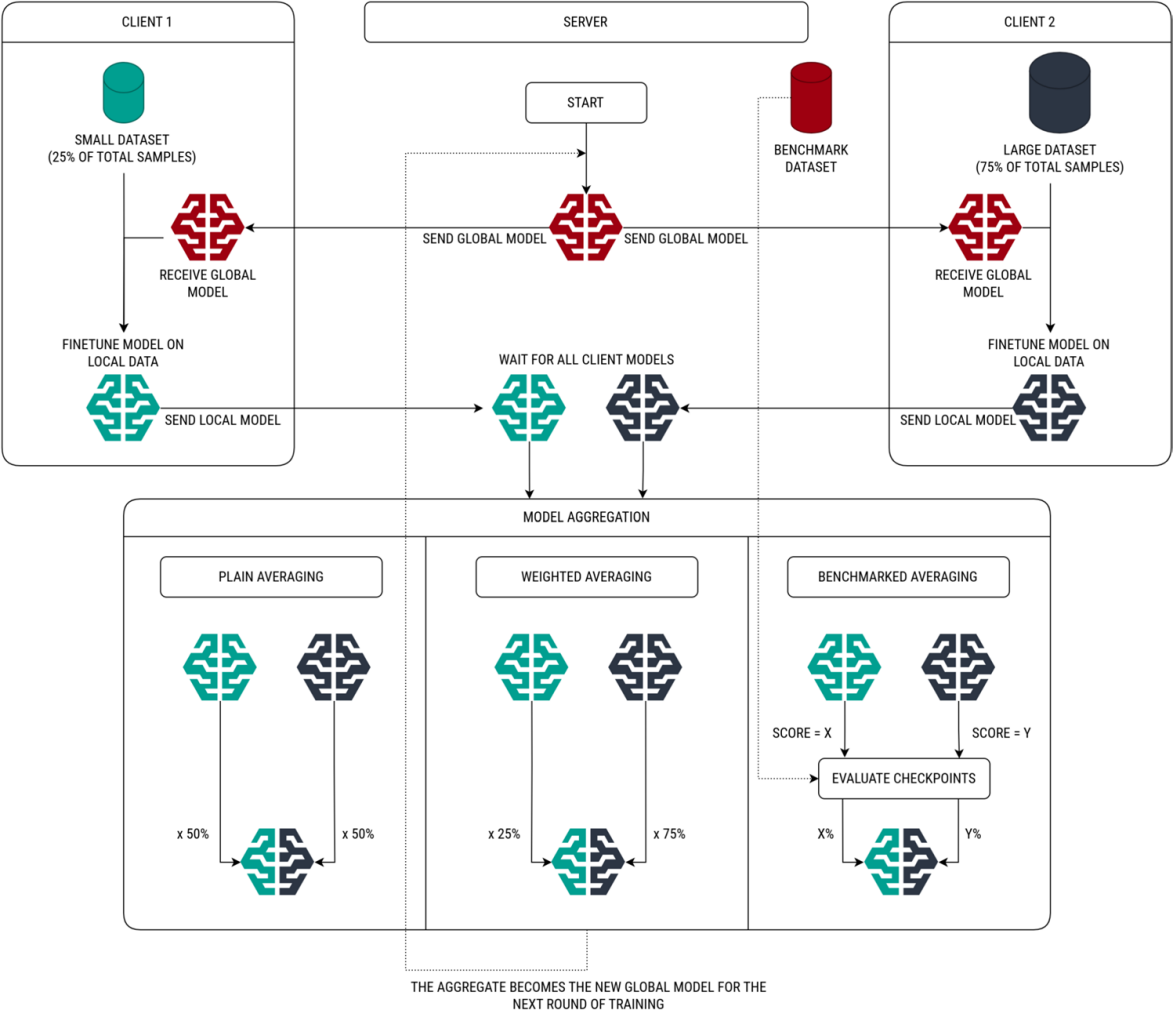
The federated learning process is initiated by the server, either by sending the latest global model to all clients, or by requesting a newly trained model in the case of the first round. Each client receives the global model and fine-tunes it using locally available data. The newly trained models are returned to the server, and once all client models are received, the server aggregates them into a new global model. This process is repeated for a predefined number of rounds. Users can choose from three aggregation strategies: plain aggregation (i.e., the checkpoints are averaged as is), weighted aggregation (i.e., the checkpoints are balanced by the proportion of data samples), and benchmarked aggregation (i.e., the weights are normalized based on the checkpoint performance on a benchmark dataset)

The aggregation strategy is an essential part of federated learning. Within the scope of federated learning experiments, three distinct averaging strategies are utilized: plain, weighted, and benchmarked averaging, as illustrated in Fig. 3. In the case of plain averaging, the checkpoints are averaged as they are, which can sometimes be a naive and non-optimal approach. For example, if particular clients utilize datasets with considerably more samples than others, their contribution should also matter more. In the case of weighted averaging, the checkpoint weights of each client are typically scaled by the ratio of dataset samples they utilize, though users can also experiment with custom ratios. However, not all datasets are of the same quality, and a benchmark dataset for server-side evaluation can be beneficial. This introduces an averaging strategy referred to as benchmarked averaging. In this approach, a benchmark dataset is utilized to evaluate the performance of checkpoints received from clients in each round. Next, a user specified metric (e.g., $ROC\text {-}AUC$ or $R^2$) is computed, and weights are adjusted according to the performance of each client. While benchmarked averaging is expected to perform better than plain and weighted averaging, it must be evaluated carefully, as effectiveness heavily depends on the quality and breadth of the benchmark dataset. If the dataset has too few samples or is not representative of the overall distribution, it can negatively impact the federated learning process. Some clients may have access to valuable but rare dataset samples that score low on the benchmark dataset and are incorrectly penalized. On the other hand, benchmarked aggregation can protect against security threats such as model poisoning or evasion [59] attacks. While dataset collection and evaluation are outside kMoL’s area of focus, Tanimoto-based diversity metrics can be used to evaluate the benchmark dataset before the federated learning process. If the dataset lacks robust coverage of the chemical space, a different aggregation method may be more appropriate.

In addition to different averaging strategies, federated learning experiments are designed to analyze the effect of different setups by modifying dataset splitting and training duration conditions. Datasets are divided among a minimum of two and up to a maximum of twenty clients. This division encompasses balanced and imbalanced division strategies, where some clients receive more data than others. Additionally, the training process duration, measured in the number of epochs, is analyzed to investigate its impact on the training outcome.

**Fig. 4:**
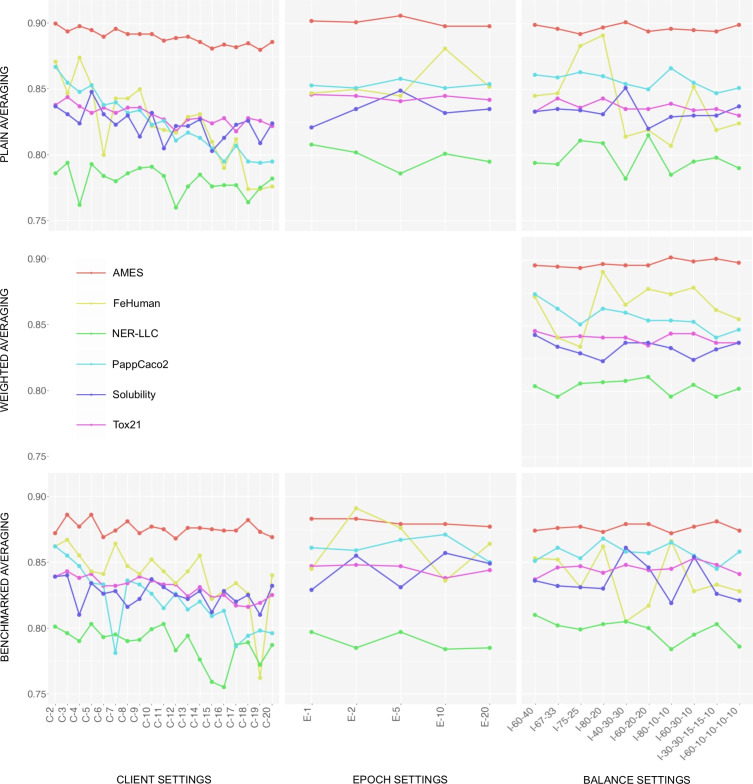
Results of federated learning classification experiments: The X-axis represents the identifier of the experiment, and the Y-axis represents the $ROC\text {-}AUC$ metric value. The client experiment settings vary in terms of the number of clients among which the data is distributed. The epoch experiment settings vary in terms of the number of epochs trained per round. The imbalance experiment settings vary in terms of the ratios of unevenly distributed data. (e.g., I-60–40 represents the setting where one client receives 60%, and the other client receives 40% of the data samples)

**Fig. 5:**
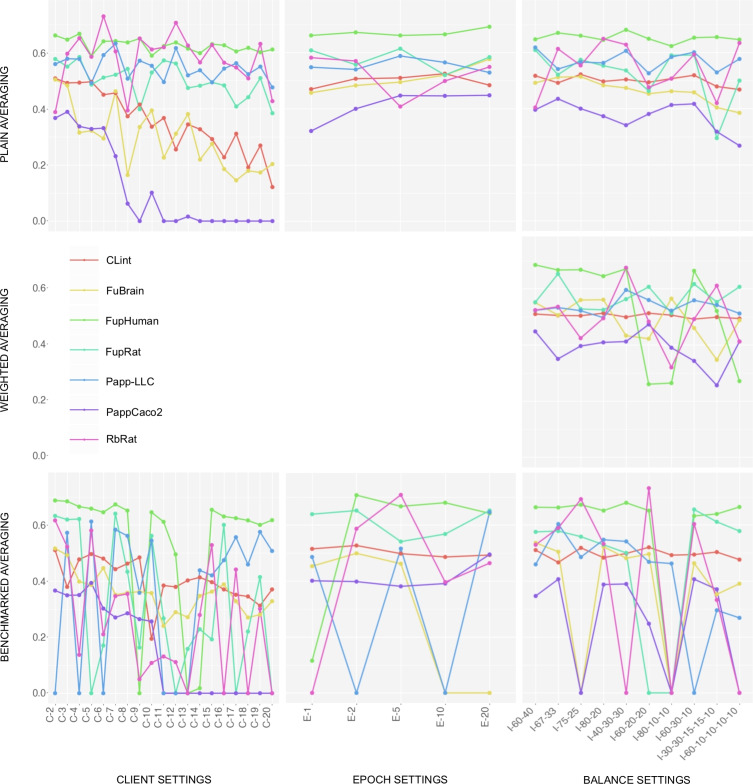
Results of federated learning regression experiments: The X-axis represents the identifier of the experiment, and the Y-axis represents the $R^2$ metric value. The client experiment settings vary in terms of the number of clients among which the data is distributed. The epoch experiment settings vary in terms of the number of epochs trained per round. The imbalance experiment settings vary in terms of the ratios of unevenly distributed data. (e.g., I-60–40 represents the setting where one client receives 60%, and the other client receives 40% of the data samples)

Federated learning experiment results are visualized in Figs. 4 and 5, and numerical values for each of the individual models are outlined in the supplementary information, section S2. In the C-X experiment settings, the data is split evenly across X clients, and models are trained for ten epochs per round. In the E-X experiment settings, the data is split between two clients, and models are trained for X epochs per round. In the I-X-Y experiment settings, models are trained for ten epochs per round, but the data is intentionally split unevenly among clients based on the specified proportions. For example, in the I-40–30-30 experiment setting, the data is split among three clients in total, where one client receives 40% of the samples and the other two clients each receive 30% of the samples. A detailed analysis of the federated learning experiment results is outlined in the Discussion section.

## Discussion

The benchmarking experiments conducted on MoleculeNet [5] datasets provide insights into the performance of the kMoL library. By utilizing Bayesian optimization for architecture search and hyperparameter tuning, kMoL demonstrated competitive results when compared to state-of-the-art models. The performance of kMoL on both classification and regression tasks underscores its ability to deliver robust machine learning models across a diverse range of tasks, from quantum mechanics and physical chemistry to biophysics and physiology. Results presented in Tables 1 and 2 show that kMoL often exceeds the performance of well-established models, making it a strong candidate for practical applications.

Results from federated learning experiments highlight several critical observations regarding the performance of the kMoL library across the different datasets and experimental conditions. One observation is that smaller datasets exhibit higher variability in metrics and are generally less reliable. For instance, splitting a dataset that contains two hundred samples among twenty clients results in just ten samples per client, leading to instability. Thus, the smaller ADME datasets require careful analysis. Over 1,000 experiments totaling more than 20,000 epochs were conducted, and while several trends were observed, there are exceptions to nearly every one of them.

A consistent trend is that the $ROC\text {-}AUC$ metric requires more time to converge as the number of clients increases, as detailed in the raw federated training results provided in the supplementary information. This occurrence is particularly noticeable in the case of the Tox21 and AMES datasets. The performance impact on the regression tasks is more pronounced than the impact on the classification tasks. For larger datasets like CLint, $R^2$ losses can exceed 50%, and convergence fails in some cases like PappCaco2. Although benchmarked aggregation shows improvement for larger datasets, it can fail in smaller or challenging datasets due to divergence in early phases. Classification tasks generally perform well across almost all tasks, with minor losses, even in cases with a large number of clients.

Occasionally, performance decreases as the number of clients increases, although not consistently. In particular scenarios, the federation process appears to act as a regularizer, similar to Stochastic Weight Averaging [75], which improves generalization. When no clear trend is observed, the differences may be due to random variations akin to local training with different random splits.

The number of epochs per round or the level of imbalance among clients does not significantly affect metrics. However, training for too many epochs (e.g., 20 epochs per round) can sometimes have adverse effects. Plain aggregation generally benefits from more rounds, while benchmarked aggregation performs better with fewer rounds.

The choice of aggregation strategy generally has limited impact, particularly for classification tasks, though specific scenarios may exhibit exceptions. Weighted averaging by sample number generally improves performance, but exceptions exist. The benchmarked aggregation, while expected to outperform naive methods, sometimes does not, especially when using more complex or small datasets. Nonetheless, it slightly outperforms other methods on classification tasks, albeit not significantly.

Comparing the federated learning results to baseline values from local training presented in Table 4, consistent trends across most experiments can be observed. Performance losses tend to increase as the number of clients the data is spread across increase, but they remain small for classification tasks. For classification tasks, losses in 2 client settings are between 0–6%, and grow to 2–8% at 20 clients. For regression tasks, the impact is more pronounced. Some experiments show moderate performance losses from 2–3% (2 clients) to 8–10% (20 clients), but there are significant losses as well, like CLint, which improves compared to the baseline by 1% in two client settings, but drops by 36% with 20 clients. Regression tasks pose greater challenges for federated learning due to their sensitivity to data variance and noise. Unlike classification tasks with simpler loss landscapes, regression requires modeling fine-grained relationships that are more impacted by fragmentation. Aggregation methods can play an important role here, with re-weighted strategies demonstrating up to 25% performance differences, as shown in supplementary information, section S2.

This work provides a foundation and practical tool for exploring and experimenting with federated learning in drug discovery, but several promising areas for further research remain. Future efforts could focus on incorporating additional public datasets of high relevance to the drug discovery community. Baselines and hyperparameters reported for ChEMBL [44], MoleculeNet [5], and TDCommons [4] datasets could serve as strong starting points for further federated learning experimentation. Aggregation strategies are another broad area of research, where dynamic weighting, clustering-based methods to group clients with similar data distributions, or gradient clipping techniques to reduce the impact of extreme updates could enhance robustness and performance in non-IID settings. [76] Regression tasks, in particular, show greater sensitivity to noise and variability across clients and may benefit from these approaches.

## Conclusion

kMoL presents a powerful and flexible solution for machine and federated learning in drug discovery. As it is developed with fast and practical experimentation in mind, the library allows users to easily configure and customize machine learning models without requiring consortium memberships, specific hardware, complicated server setups, or additional programming obligations. This flexibility makes it accessible to users from various backgrounds, from beginner researchers to seasoned professionals.

kMoL provides several mechanisms to address different types of bias during federated learning. These can be grouped into local training strategies, checkpoint aggregation strategies, and post-training strategies. Local training options include regularization techniques, dropout adjustments, and adjusting the number of training epochs per round to control how far clients deviate from the global checkpoint, whether to maintain closer alignment or allow greater divergence as needed. Checkpoint aggregation strategies are server-side approaches where users can adjust the contribution of each client based on dataset size or checkpoint quality to address imbalances. Post-training methods, such as fine-tuning the global checkpoint on a separate dataset, provide opportunities to further refine the model or correct residual bias. These tools and features are integrated into kMoL and can be applied without requiring custom code, making the framework practical for diverse scenarios.

The evaluation across various datasets demonstrates both the strengths and limitations of the kMoL library and the underlying technologies. Utilizing Bayesian Optimization for architecture searching and hyperparameter tuning enables users to rapidly and automatically find the best-performing model for a particular task. While various federated learning strategies are already enabled within the library, users are ultimately encouraged to run varied experiments on their setups to determine what works best, as no single strategy consistently excels in all scenarios.

## Supplementary information

Supplementary material 1. The supplementary information for this research project consists of two additional files, which contain the following information: The optimal values of the benchmarking experiment model hyperparameters. The results of the federated learning regression and classification experiments using the plain, weighted, and benchmarked averaging strategy. The results of the additional experiments using the TDCommons [4] dataset. The results of the additional experiments using the ChEMBL [44] dataset. Round-by-round federated training results presented in tabular format across multiple metrics.

Supplementary material 2.

## Data Availability

The kMoL library and all relevant datasets, configuration files, scripts, Docker files, and documentation are available on GitHub under the MIT open-source license at https://github.com/elix-tech/kmol.

## References

[1] Dara S, Dhamercherla S, Jadav SS, Babu CM, Ahsan MJ (2022) Machine learning in drug discovery: A review. Artif Intell Rev 55(3):1947–1999. 10.1007/s10462-021-10058-4 34393317 10.1007/s10462-021-10058-4 PMC8356896

[2] Ma J, Sheridan RP, Liaw A, Dahl GE, Svetnik V (2015) Deep neural nets as a method for quantitative structure-activity relationships. J Chem Inf Model 55(2):263–274. 10.1021/ci500747n 25635324 10.1021/ci500747n

[3] Xu Y, Ma J, Liaw A, Sheridan RP, Svetnik V (2017) Demystifying multitask deep neural networks for quantitative structure-activity relationships. J Chem Inf Model 57(10):2490–2504. 10.1021/acs.jcim.7b00087 28872869 10.1021/acs.jcim.7b00087

[4] Huang K, Fu T, Gao W, Zhao Y, Roohani Y, Leskovec J, Coley CW, Xiao C, Sun J, Zitnik M (2021) Therapeutics data commons: Machine learning datasets and tasks for drug discovery and development. Proceedings of Neural Information Processing Systems, NeurIPS Datasets and Benchmarks

[5] Wu Z, Ramsundar B, Feinberg EN, Gomes J, Geniesse C, Pappu AS, Leswing K, Pande V (2018) MoleculeNet: A Benchmark for Molecular Machine Learning 10.1039/c7sc02664a PMC5868307 29629118

[6] Paracha A, Arshad J, Farah MB, Ismail K (2024) Machine learning security and privacy: a review of threats and countermeasures. EURASIP J Inf Secur 2024(1):10. 10.1186/s13635-024-00158-3

[7] Heyndrickx W, Mervin L, Morawietz T, Sturm N, Friedrich L, Zalewski A, Pentina A, Humbeck L, Oldenhof M, Niwayama R, Schmidtke P, Fechner N, Simm J, Arany A, Drizard N, Jabal R, Afanasyeva A, Loeb R, Verma S, Harnqvist S, Holmes M, Pejo B, Telenczuk M, Holway N, Dieckmann A, Rieke N, Zumsande F, Clevert D-A, Krug M, Luscombe C, Green D, Ertl P, Antal P, Marcus D, Do Huu N, Fuji H, Pickett S, Acs G, Boniface E, Beck B, Sun Y, Gohier A, Rippmann F, Engkvist O, Göller AH, Moreau Y, Galtier MN, Schuffenhauer A, Ceulemans H (2024) Melloddy: Cross-pharma federated learning at unprecedented scale unlocks benefits in qsar without compromising proprietary information. J Chem Inf Model 64(7):2331–2344. 10.1021/acs.jcim.3c00799 37642660 10.1021/acs.jcim.3c00799 PMC11005050

[8] NVIDIA: NVIDIA Clara. Accessed: 2024-08-26. https://docs.nvidia.com/clara/index.html

[9] Beutel DJ, Topal T, Mathur A, Qiu X, Fernandez-Marques J, Gao Y, Sani L, Kwing HL, Parcollet T, Gusmão PPd, Lane ND (2020) Flower: A friendly federated learning research framework. arXiv preprint arXiv:2007.14390

[10] Liu Y, Fan T, Chen T, Xu Q, Yang Q (2021) Fate: an industrial grade platform for collaborative learning with data protection. J Mach Learn Res 22:1

[11] Ziller A, Trask A, Lopardo A, Szymkow B, Wagner B, Bluemke E, Nounahon J-M, Passerat-Palmbach J, Prakash K, Rose N, Ryffel T, Reza ZN, Kaissis G (2021) PySyft A Library for Easy Federated Learning. Springer, Cham

[12] Chen S, Xue D, Chuai G, Yang Q, Liu Q (2020) Fl-qsar: a federated learning-based qsar prototype for collaborative drug discovery. Bioinformatics 36(22–23):5492–5498 10.1093/bioinformatics/btaa1006 33289524

[13] Guo Y, Gao Y, Song J (2024) Molcfl: A personalized and privacy-preserving drug discovery framework based on generative clustered federated learning. J Biomed Inf 157:104712. 10.1016/j.jbi.2024.104712 10.1016/j.jbi.2024.104712 39182631

[14] ...Oldenhof M, Ács G, Pejó B, Schuffenhauer A, Holway N, Sturm N, Dieckmann A, Fortmeier O, Boniface E, Mayer C, Gohier A, Schmidtke P, Niwayama R, Kopecky D, Mervin L, Rathi PC, Friedrich L, Formanek A, Antal P, Rahaman J, Zalewski A, Heyndrickx W, Oluoch E, Stößel M, Vančo M, Endico D, Gelus F, Boisfossé T, Darbier A, Nicollet A, Blottière M, Telenczuk M, Nguyen VT, Martinez T, Boillet C, Moutet K, Picosson A, Gasser A, Djafar I, Simon A, Arany Á, Simm J, Moreau Y, Engkvist O, Ceulemans H, Marini C, Galtier M (2024) Industry-scale orchestrated federated learning for drug discovery. Proc AAAI Conf Artif Intell 37(13):15576–15584. 10.1609/aaai.v37i13.26847

[15] Landrum G RDKit: Open-source cheminformatics. http://www.rdkit.org

[16] Todeschini, R., VC, Wang, R, YF, Ghose, AK, GC, Sharma, V, RG, Stanton, DT, PJ, Yap C, Cao, D-S, Q-SX, Cao, D-S, Y-ZL, Cao, D-S, NX, O’Boyle, NM, GH al (1970) Mordred: a molecular descriptor calculator. Journal of Cheminformatics

[17] Rogers D, Hahn M (2010) Extended-connectivity fingerprints. J Chem Inf Model 50(5):742–754. 10.1021/ci100050t. 20426451 10.1021/ci100050t

[18] Butina D (1999) Unsupervised data base clustering based on daylight’s fingerprint and tanimoto similarity: A fast and automated way to cluster small and large data sets. J Chem Inf Comput Sci 39(4):747–750. 10.1021/ci9803381

[19] Sundararajan M, Taly A, Yan Q (2017) Axiomatic Attribution for Deep Networks

[20] Kokhlikyan N, Miglani V, Martin M, Wang E, Alsallakh B, Reynolds J, Melnikov A, Kliushkina N, Araya C, Yan S, Reblitz-Richardson O (2020) Captum: A unified and generic model interpretability library for PyTorch

[21] Paszke A, Gross S, Massa F, Lerer A, Bradbury J, Chanan G, Killeen T, Lin Z, Gimelshein N, Antiga L, Desmaison A, Kopf A, Yang E, DeVito Z, Raison M, Tejani A, Chilamkurthy S, Steiner B, Fang L, Bai J, Chintala S (2019) Pytorch: An imperative style, high-performance deep learning library. In: Wallach, H., Larochelle, H., Beygelzimer, A., Alché-Buc, F., Fox, E., Garnett, R. (eds.) Advances in Neural Information Processing Systems 32, pp. 8024–8035. Curran Associates, Inc., Red Hook, NY. http://papers.neurips.cc/paper/9015-pytorch-an-imperative-style-high-performance-deep-learning-library.pdf

[22] Fey M, Lenssen JE (2019) Fast Graph Representation Learning with PyTorch Geometric

[23] Thomas N Kipf, MW (2017) Semi-supervised classification with graph convolutional networks. Arxiv

[24] Gilmer J, Schoenholz SS, Riley PF, Vinyals O, Dahl GE (2017) Neural Message Passing for Quantum Chemistry

[25] Defferrard M, Bresson X, Vandergheynst P (2017) Convolutional Neural Networks on Graphs with Fast Localized Spectral Filtering

[26] Hamilton WL, Ying R, Leskovec J (2018) Inductive Representation Learning on Large Graphs

[27] Morris C, Ritzert M, Fey M, Hamilton WL, Lenssen JE, Rattan G, Grohe M (2020) Weisfeiler and Leman Go Neural: Higher-order Graph Neural Networks

[28] Xu K, Hu W, Leskovec J, Jegelka S (2019) How Powerful are Graph Neural Networks?

[29] Bianchi FM, Grattarola D, Livi L, Alippi C (2021) Graph Neural Networks with convolutional ARMA filters 10.1109/TPAMI.2021.3054830 33497331

[30] Ranjan E, Sanyal S, Talukdar PP (2020) ASAP: Adaptive Structure Aware Pooling for Learning Hierarchical Graph Representations

[31] Li G, Xiong C, Thabet A, Ghanem B (2020) DeeperGCN: All You Need to Train Deeper GCNs

[32] Chiang W-L, Liu X, Si S, Li Y, Bengio S, Hsieh C-J (2019) Cluster-gcn: An efficient algorithm for training deep and large graph convolutional networks. Proceedings of the 25th ACM SIGKDD International Conference on Knowledge Discovery & Data Mining10.1145/3292500.3330925

[33] Verma N, Boyer E, Verbeek J (2018) FeaStNet: Feature-Steered Graph Convolutions for 3D Shape Analysis

[34] Veličković P, Cucurull G, Casanova A, Romero A, Liò P, Bengio Y (2018) Graph Attention Networks

[35] Du J, Zhang S, Wu G, Moura JMF, Kar S (2018) Topology Adaptive Graph Convolutional Networks

[36] Wu F, Zhang T, Souza Jr. au2 AH, Fifty C, Yu T, Weinberger KQ (2019) Simplifying Graph Convolutional Networks

[37] Li P, Li Y, Hsieh C-Y, Zhang S, Liu X, Liu H, Song S, Yao X (2020) TrimNet: learning molecular representation from triplet messages for biomedicine. Brief Bioinform. 10.1093/bib/bbaa266/34130210/bbaa266.pdf 33147620 10.1093/bib/bbaa266

[38] He K, Zhang X, Ren S, Sun J (2015) Deep Residual Learning for Image Recognition

[39] Ioffe S, Szegedy C (2015) Batch Normalization: Accelerating Deep Network Training by Reducing Internal Covariate Shift

[40] Ulyanov D, Vedaldi A, Lempitsky V (2017) Instance Normalization: The Missing Ingredient for Fast Stylization

[41] Cai T, Luo S, Xu K, He D, Liu T-Y, Wang L (2021) GraphNorm: A Principled Approach to Accelerating Graph Neural Network Training

[42] Srivastava N, Hinton G, Krizhevsky A, Sutskever I, Salakhutdinov R (2014) Dropout: A simple way to prevent neural networks from overfitting. J Mach Learn Res 15(56):1929–1958

[43] Agarap AF (2019) Deep Learning using Rectified Linear Units (ReLU)

[44] Gaulton A, Hersey A, Nowotka M, Bento AP, Chambers J, Mendez D, Mutowo P, Atkinson F, Bellis LJ, Cibrián-Uhalte E, Davies M, Dedman N, Karlsson A, Magariños MP, Overington JP, Papadatos G, Smit I, Leach AR (2016) The chembl database in 2017. Nucleic Acids Res 45(D1):945–954 10.1093/nar/gkw1074 PMC5210557 27899562

[45] Amini A, Schwarting W, Soleimany A, Rus D (2020) Deep Evidential Regression. https://arxiv.org/abs/1910.02600

[46] Sensoy M, Kaplan L, Kandemir M (2018) Evidential Deep Learning to Quantify Classification Uncertainty. https://arxiv.org/abs/1806.01768

[47] Zhuang J, Tang T, Ding Y, Tatikonda S, Dvornek N, Papademetris X, Duncan JS (2020) AdaBelief Optimizer: Adapting Stepsizes by the Belief in Observed Gradients

[48] Bergstra J, Bardenet R, Bengio Y, Kégl B (2011) Algorithms for hyper-parameter optimization. In: Proceedings of the 24th International Conference on Neural Information Processing Systems. NIPS’11, pp. 2546–2554. Curran Associates Inc., Red Hook, NY, USA

[49] Bergstra J, Yamins D, Cox D (2013) Making a science of model search: Hyperparameter optimization in hundreds of dimensions for vision architectures. In: Dasgupta, S., McAllester, D. (eds.) Proceedings of the 30th International Conference on Machine Learning. Proceedings of Machine Learning Research, vol. 28, pp. 115–123. PMLR, Atlanta, Georgia, USA. http://proceedings.mlr.press/v28/bergstra13.html

[50] Akiba T, Sano S, Yanase T, Ohta T, Koyama M (2019) Optuna: A Next-generation Hyperparameter Optimization Framework

[51] Gal Y, Ghahramani Z (2016) Dropout as a bayesian approximation: Representing model uncertainty in deep learning. In: Balcan, M.F., Weinberger, K.Q. (eds.) Proceedings of The 33rd International Conference on Machine Learning. Proceedings of Machine Learning Research, vol. 48, pp. 1050–1059. PMLR, New York, New York, USA. https://proceedings.mlr.press/v48/gal16.html

[52] Sensoy M, Kaplan L, Kandemir M (2018) Evidential Deep Learning to Quantify Classification Uncertainty. https://arxiv.org/abs/1806.01768

[53] Amini A, Schwarting W, Soleimany A, Rus D (2020) Deep Evidential Regression. https://arxiv.org/abs/1910.02600

[54] gRPC: gRPC - An RPC library and framework. Accessed: 2024-08-26. https://grpc.io

[55] Box I Box: The Intelligent Content Cloud. https://www.box.com

[56] Dwork C, Roth A (2014) The algorithmic foundations of differential privacy. Found Trends Theor Comput Sci 9(3–4):211–407. 10.1561/0400000042

[57] Dwork C, Roth A (2014) The Algorithmic Foundations of Differential Privacy. now Publishers Inc, Boston

[58] Opacus PyTorch library. Available from https://opacus.ai

[59] Rigaki M, Garcia S (2020) A Survey of Privacy Attacks in Machine Learning

[60] Huang R, Xia M, Nguyen D-T, Zhao T, Sakamuru S, Zhao J, Shahane S, Rossoshek A, Simeonov A (2016) Tox21challenge to build predictive models of nuclear receptor and stress response pathways as mediated by exposure to environmental chemicals and drugs. Front Environ Sci. 10.3389/fenvs.2015.00085

[61] Hansen K, Mika S, Schroeter T, Sutter A, Laak A, Steger-Hartmann T, Heinrich N, Müller K-R (2009) Benchmark data set for in silico prediction of ames mutagenicity. J Chem Inf Model 49(9):2077–2081. 10.1021/ci900161g 19702240 10.1021/ci900161g

[62] Ames Conclusions Data Collection. Accessed: 2020-12-02 (2020). ftp://anonftp.niehs.nih.gov/ntp-cebs/datatype/NTP_Data_Collections/Ames_Conclusions_DataCollection_2020-02-19.xlsx

[63] PubChem Bioassay Record for AID 1259408, GENE-TOX mutagenicity studies. Accessed: 2024-9-25. https://pubchem.ncbi.nlm.nih.gov/bioassay/1259408

[64] Kawashima H, Watanabe R, Esaki T, Kuroda M, Nagao C, Natsume-Kitatani Y, Ohashi R, Komura H, Mizuguchi K (2023) Drumap: A novel drug metabolism and pharmacokinetics analysis platform. J Med Chem 66(14):9697–9709. 10.1021/acs.jmedchem.3c00481 37449459 10.1021/acs.jmedchem.3c00481 PMC10388294

[65] Fang X, Liu L, Lei J, He D, Zhang S, Zhou J, Wang F, Wu H, Wang H (2022) Geometry-enhanced molecular representation learning for property prediction. Nat Mach Intell 4(2):127–134. 10.1038/s42256-021-00438-4

[66] Zhou G, Gao Z, Ding Q, Zheng H, Xu H, Wei Z, Zhang L, Ke G (2023) Uni-mol: A universal 3d molecular representation learning framework. In: The Eleventh International Conference on Learning Representations. https://openreview.net/forum?id=6K2RM6wVqKu

[67] Han X, Jia M, Chang Y, Li Y, Wu S (2022) Directed message passing neural network (d-mpnn) with graph edge attention (gea) for property prediction of biofuel-relevant species. Energy AI 10:100201. 10.1016/j.egyai.2022.100201

[68] Xiong Z, Wang D, Liu X, Zhong F, Wan X, Li X, Li Z, Luo X, Chen K, Jiang H, Zheng M (2020) Pushing the boundaries of molecular representation for drug discovery with the graph attention mechanism. J Med Chem 63(16):8749–8760. 10.1021/acs.jmedchem.9b00959 31408336 10.1021/acs.jmedchem.9b00959

[69] Liu S, Demirel MF, Liang Y (2019) N-Gram Graph: Simple Unsupervised Representation for Graphs, with Applications to Molecules. https://arxiv.org/abs/1806.09206

[70] Hu W, Liu B, Gomes J, Zitnik M, Liang P, Pande V, Leskovec J (2020) Strategies for Pre-training Graph Neural Networks. https://arxiv.org/abs/1905.12265

[71] Rong Y, Bian Y, Xu T, Xie W, Wei Y, Huang W, Huang J (2020) Self-Supervised Graph Transformer on Large-Scale Molecular Data. https://arxiv.org/abs/2007.02835

[72] Liu S, Wang H, Liu W, Lasenby J, Guo H, Tang J (2022) Pre-training Molecular Graph Representation with 3D Geometry. https://arxiv.org/abs/2110.07728

[73] Wang Y, Wang J, Cao Z, Barati Farimani A (2022) Molecular contrastive learning of representations via graph neural networks. Nat Mach Intell 4(3):279–287. 10.1038/s42256-022-00447-x

[74] Xia J, Zhao C, Hu B, Gao Z, Tan C, Liu Y, Li S, Li SZ (2023) Mole-BERT: Rethinking pre-training graph neural networks for molecules. In: The Eleventh International Conference on Learning Representations. https://openreview.net/forum?id=jevY-DtiZTR

[75] Izmailov P, Podoprikhin D, Garipov T, Vetrov D, Wilson AG (2019) Averaging Weights Leads to Wider Optima and Better Generalization

[76] Xiong Z, Cheng Z, Lin X, Xu C, Liu X, Wang D, Luo X, Zhang Y, Jiang H, Qiao N, Zheng M (2022) Facing small and biased data dilemma in drug discovery with enhanced federated learning approaches. Sci China Life Sci 65(3):529–539. 10.1007/s11427-021-1946-0 34319533 10.1007/s11427-021-1946-0

