# Supplementary material for: kMoL: an open-source machine and federated learning library for drug discovery
Source: J Cheminform. 2025 Feb 25;17:22. doi: 10.1186/s13321-025-00967-9 (PMC11854109; doi:10.1186/s13321-025-00967-9)
Supplement: Supplementary file 2 — Supplementary material 2. [file 13321_2025_967_MOESM2_ESM.pdf]

Supplementary Information for kMoL: An Open-source Machine and Federated Learning Library for Drug Discovery

Romeo Cozac[1*], Haris Hasic[1], Jun Jin Choong[1], Vincent Richard[1], Loic Beheshti[1], Cyrille Froehlich[1], Takuto Koyama[2], Shigeyuki Matsumoto[2], Ryosuke Kojima[2], Hiroaki Iwata[2], Aki Hasegawa[2], Takao Otsuka[2], Yasushi Okuno[2*]

[1*]Elix, Inc., 8-34 Yonbancho, Chiyoda-ku, Tokyo 102-0081, Japan.
[2*]Graduate School of Medicine, Kyoto University, Shogoin-kawaharacho, Sakyo-ku, Kyoto 606-8507, Japan.

*Corresponding author(s). E-mail(s): romeo.cozac@elix-inc.com; okuno.yasushi.4c@kyoto-u.ac.jp;

S1 Model hyperparameters

In benchmarking experiments, the following hyperparameters were tuned using Bayesian optimization for a total of 25 trials: graph layer type, number of graph layers (2 to 8), number of hidden features (32 to 256), dropout rate (0.0 to 0.7), optimizer type (Stochastic Gradient Descent (SGD) [1], Adam [2], AdamW [3], or AdaBelief [4]), learning rate (0.0001 to 0.01), weight decay rate (0.00001 to 0.001), and utilization of residual layers (Yes or No). The results are presented in Supplementary Table 1.

In federated learning experiments, the following hyperparameters were tuned using Bayesian optimization for a total of 500 trials: graph layer type, number of graph layers (2 to 12), number of hidden features (32 to 256), dropout rate (0.0 to 0.7), optimizer type (SGD [1], Adam [2], AdamW [3], or AdaBelief [4]), learning rate (0.0001 to 0.01), weight decay rate (0.00001 to 0.001), utilization of residual layers (Yes or No), normalization layer type (BatchNorm [5], LayerNorm [6], or GraphNorm [7]), utilization of edge features (Yes or No), and utilization of molecular-level features (Yes or No). The results are presented in Supplementary Table 2 excluding the utilization of residual layers and molecular-level features values which were Yes for every model.

Supplementary Table 1 Optimal values for benchmarking experiment model hyperparameters.

Dataset	Layer Type	Layers	Hidden Features	Dropout	Optimizer	Learning Rate	Weight Decay	Residual
SIDER	GraphConv [8]	6	256	0	Adam [2]	0.01	0.00001	No
ClinTox	GINConv [9]	7	144	0.3	AdaBelief [4]	0.001	0.00076	Yes
BACE	GINConv [9]	5	64	0.1	AdaBelief [4]	0.0066	0.00042	No
BBBP	GINConv [9]	6	80	0	AdamW [3]	0.0088	0.00078	Yes
Tox21	LEConv [10]	7	96	0.1	AdamW [3]	0.01	5.6e-4	No
Toxcast	LEConv [10]	7	176	0	AdamW [3]	0.0099	0.001	No
HIV	SAGEConv [11]	4	160	0.1	AdamW [3]	0.0021	0.00071	No
MUV	LEConv [10]	5	112	0	SGD [1]	0.0033	5e-05	No
PCBA	GINConv [9]	7	240	0.3	AdamW [3]	0.0085	0.00013	No
FreeSolv	GraphConv [8]	7	240	0	AdaBelief [4]	0.0086	0.0006	No
ESOL	GINConv [9]	3	144	0.1	AdaBelief [4]	0.005	0.00026	No
Lipophilicity	GCNConv [12]	4	208	0	AdaBelief [4]	0.0033	0.00089	Yes
QM7	GCNConv [12]	4	96	0	AdamW [3]	0.005	0.00016	No
QM8	SAGEConv [11]	6	176	0	AdaBelief [4]	0.0013	0.0002	Yes

Supplementary Table 2 Optimal values for federated learning experiment model hyperparameters.

Dataset	Layer Type	Layers	Hidden Features	Dropout	Optimizer	Learning Rate	Weight Decay	Normalization	Edge Features
Tox21	LEConv [10]	7	96	0.1	AdamW [3]	0.01	5.6e-4	Batch [5]	No
AMES	GCNConv [12]	3	160	0.1	AdaBelief [4]	0.01	3.6e-4	Batch [5]	No
CLint	GENConv [13]	2	128	0.1	AdamW [3]	0.01	6e-6	Batch [5]	Yes
FeHuman	GCNConv [12]	4	64	0.2	AdamW [3]	0.003	5e-4	Batch [5]	No
FuBrain	TrimConv [14]	3	64	0.2	AdamW [3]	0.0045	1.6e-6	Batch [5]	Yes
FupHuman	GINConv [9]	2	128	0.15	AdamW [3]	0.003	1.5e-6	Batch [5]	No
FupRat	GENConv [13]	3	64	0.1	AdamW [3]	0.01	2e-6	Layer [6]	No
NER-LLC	GINConv [9]	2	32	0.25	AdamW [3]	0.01	1e-4	Graph [7]	No
PappCaco2	GINConv [9]	4	128	0.1	AdamW [3]	0.0008	2e-4	Batch [5]	No
Papp-LLC	GCNConv [12]	2	32	0.2	AdamW [3]	0.01	8e-6	Batch [5]	No
RbRat	GCNConv [12]	3	32	0.15	AdamW [3]	0.017	1e-5	Batch [5]	Yes
Solubility	GINConv [9]	2	128	0.1	AdamW [3]	0.001	5e-5	Batch [5]	No

S2 Federated learning experiments

Accompanying Figures 3 and 4 from the main manuscript, the same data is presented in numeric tabular format in this section. A "Baseline" row at the head of the table represents local training results, where all data is trained in the same process. These baseline values match those presented in Table 4 from the main manuscript.

Supplementary Table 3 Results for federated learning classification experiments utilizing plain averaging strategy reported using the *ROC-AUC* metric.

Setting	Tox21	AMES	FeHuman	NER-LLC	PappCaco2	Solubility
Baseline	0.869	0.903	0.921	0.821	0.860	0.861
C-2	0.838	0.900	0.871	0.786	0.867	0.837
C-3	0.844	0.894	0.847	0.794	0.855	0.831
C-4	0.837	0.898	0.874	0.762	0.848	0.824
C-5	0.832	0.895	0.853	0.793	0.853	0.848
C-6	0.836	0.890	0.800	0.784	0.838	0.831
C-7	0.832	0.896	0.843	0.780	0.840	0.823
C-8	0.836	0.892	0.843	0.786	0.832	0.830
C-9	0.836	0.892	0.850	0.790	0.834	0.814
C-10	0.831	0.892	0.822	0.791	0.823	0.832
C-11	0.827	0.887	0.819	0.784	0.826	0.805
C-12	0.818	0.889	0.817	0.760	0.811	0.822
C-13	0.827	0.890	0.829	0.776	0.817	0.822
C-14	0.828	0.886	0.831	0.785	0.813	0.827
C-15	0.824	0.881	0.810	0.776	0.805	0.803
C-16	0.828	0.884	0.790	0.777	0.795	0.813
C-17	0.818	0.882	0.812	0.777	0.807	0.823
C-18	0.828	0.885	0.774	0.764	0.795	0.826
C-19	0.826	0.880	0.774	0.775	0.794	0.809
C-20	0.822	0.886	0.776	0.782	0.795	0.824
E-1	0.846	0.902	0.847	0.808	0.853	0.821
E-2	0.845	0.901	0.850	0.802	0.851	0.835
E-5	0.841	0.906	0.845	0.786	0.858	0.849
E-10	0.845	0.898	0.881	0.801	0.851	0.832
E-20	0.842	0.898	0.852	0.795	0.854	0.835
I-60-40	0.833	0.899	0.845	0.794	0.861	0.833
I-67-33	0.843	0.896	0.847	0.793	0.859	0.835
I-75-25	0.836	0.892	0.883	0.811	0.863	0.834
I-80-20	0.843	0.897	0.891	0.809	0.860	0.831
I-40-30-30	0.835	0.901	0.814	0.782	0.854	0.851
I-60-20-20	0.835	0.894	0.819	0.815	0.850	0.820
I-80-10-10	0.839	0.896	0.807	0.785	0.866	0.829
I-60-30-10	0.834	0.895	0.852	0.795	0.855	0.830
I-30-30-15-15-10	0.835	0.894	0.819	0.798	0.847	0.830
I-60-10-10-10-10	0.830	0.899	0.824	0.790	0.851	0.837

Supplementary Table 4 Results for federated learning regression experiments utilizing plain averaging strategy reported using the R^2 metric.

Setting	CLint	FuBrain	FupHuman	FupRat	PappCaco2	Papp-LLC	RbRat
Baseline	0.489	0.591	0.690	0.594	0.451	0.577	0.669
C-2	0.508	0.504	0.661	0.577	0.367	0.559	0.388
C-3	0.492	0.483	0.646	0.550	0.389	0.578	0.596
C-4	0.493	0.315	0.667	0.584	0.337	0.577	0.651
C-5	0.496	0.322	0.588	0.486	0.328	0.492	0.585
C-6	0.450	0.294	0.640	0.511	0.331	0.591	0.729
C-7	0.456	0.462	0.641	0.521	0.231	0.632	0.604
C-8	0.373	0.164	0.636	0.542	0.062	0.507	0.394
C-9	0.415	0.335	0.650	0.398	-0.187	0.571	0.650
C-10	0.336	0.394	0.589	0.529	0.101	0.553	0.611
C-11	0.367	0.226	0.623	0.572	-0.134	0.495	0.619
C-12	0.255	0.311	0.636	0.561	-0.141	0.616	0.706
C-13	0.344	0.381	0.614	0.474	0.016	0.519	0.625
C-14	0.327	0.219	0.598	0.482	-0.067	0.537	0.565
C-15	0.292	0.275	0.630	0.494	-0.251	0.494	0.627
C-16	0.227	0.185	0.626	0.483	-0.213	0.543	0.564
C-17	0.311	0.145	0.604	0.408	-0.165	0.562	0.547
C-18	0.191	0.179	0.617	0.441	-0.161	0.523	0.508
C-19	0.269	0.173	0.601	0.509	-0.277	0.550	0.631
C-20	0.121	0.203	0.611	0.384	-0.111	0.476	0.427
E-1	0.470	0.457	0.661	0.608	0.321	0.548	0.582
E-2	0.507	0.483	0.672	0.557	0.400	0.540	0.570
E-5	0.510	0.495	0.661	0.614	0.447	0.588	0.408
E-10	0.525	0.520	0.665	0.520	0.446	0.565	0.499
E-20	0.484	0.577	0.692	0.584	0.448	0.529	0.549
I-60-40	0.517	0.492	0.647	0.609	0.396	0.618	0.404
I-67-33	0.492	0.512	0.670	0.521	0.435	0.541	0.613
I-75-25	0.522	0.514	0.660	0.574	0.400	0.566	0.554
I-80-20	0.497	0.483	0.645	0.553	0.373	0.563	0.649
I-40-30-30	0.504	0.474	0.681	0.536	0.341	0.606	0.628
I-60-20-20	0.494	0.453	0.649	0.463	0.381	0.526	0.476
I-80-10-10	0.507	0.462	0.623	0.590	0.413	0.584	0.507
I-60-30-10	0.519	0.458	0.653	0.591	0.417	0.601	0.593
I-30-30-15-15-10	0.479	0.404	0.655	0.295	0.318	0.529	0.420
I-60-10-10-10-10	0.468	0.385	0.646	0.500	0.268	0.577	0.634

Supplementary Table 5 Results for federated learning classification experiments utilizing weighted averaging strategy reported using the *ROC-AUC* metric.

**Setting**	**Tox21**	**AMES**	**FeHuman**	**NER-LLC**	**PappCaco2**	**Solubility**
Baseline	0.869	0.903	0.921	0.821	0.860	0.861
I-60-40	0.846	0.896	0.872	0.804	0.874	0.843
I-67-33	0.841	0.895	0.841	0.796	0.863	0.834
I-75-25	0.842	0.894	0.834	0.806	0.851	0.829
I-80-20	0.841	0.897	0.891	0.807	0.863	0.823
I-40-30-30	0.841	0.896	0.866	0.808	0.860	0.837
I-60-20-20	0.835	0.896	0.878	0.811	0.854	0.837
I-80-10-10	0.844	0.902	0.874	0.796	0.854	0.833
I-60-30-10	0.844	0.899	0.879	0.805	0.853	0.824
I-30-30-15-15-10	0.837	0.901	0.862	0.796	0.841	0.832
I-60-10-10-10-10	0.837	0.898	0.855	0.802	0.847	0.837

Supplementary Table 6 Results for federated learning regression experiments utilizing weighted averaging strategy reported using the R^2 metric.

**Setting**	**CLint**	**FuBrain**	**FupHuman**	**FupRat**	**PappCaco2**	**Papp-LLC**	**RbRat**
Baseline	0.489	0.591	0.690	0.594	0.451	0.577	0.669
I-60-40	0.510	0.551	0.685	0.552	0.448	0.523	0.524
I-67-33	0.505	0.506	0.667	0.653	0.350	0.533	0.536
I-75-25	0.504	0.560	0.668	0.528	0.396	0.522	0.424
I-80-20	0.513	0.561	0.645	0.525	0.409	0.497	0.495
I-40-30-30	0.499	0.433	0.671	0.563	0.412	0.596	0.675
I-60-20-20	0.513	0.422	0.260	0.607	0.473	0.560	0.483
I-80-10-10	0.506	0.565	0.264	0.513	0.390	0.522	0.320
I-60-30-10	0.492	0.460	0.664	0.617	0.343	0.559	0.492
I-30-30-15-15-10	0.499	0.347	0.521	0.553	0.256	0.542	0.611
I-60-10-10-10-10	0.494	0.488	0.271	0.607	0.412	0.512	0.412

Supplementary Table 7 Results for federated learning classification experiments utilizing benchmarked averaging strategy reported using the *ROC-AUC* metric.

**Setting**	**Tox21**	**AMES**	**FeHuman**	**NER-LLC**	**PappCaco2**	**Solubility**
Baseline	0.869	0.903	0.921	0.821	0.860	0.861
C-2	0.839	0.872	0.862	0.801	0.862	0.839
C-3	0.843	0.886	0.867	0.796	0.855	0.840
C-4	0.838	0.877	0.855	0.790	0.847	0.810
C-5	0.841	0.886	0.843	0.803	0.834	0.834
C-6	0.832	0.869	0.841	0.793	0.833	0.826
C-7	0.832	0.874	0.864	0.795	0.781	0.828
C-8	0.834	0.881	0.847	0.790	0.836	0.816
C-9	0.839	0.872	0.841	0.791	0.833	0.822
C-10	0.836	0.877	0.852	0.799	0.826	0.837
C-11	0.833	0.875	0.843	0.803	0.815	0.831
C-12	0.833	0.868	0.834	0.783	0.826	0.825
C-13	0.824	0.876	0.843	0.794	0.814	0.822
C-14	0.831	0.876	0.855	0.776	0.820	0.828
C-15	0.823	0.875	0.822	0.759	0.809	0.812
C-16	0.825	0.874	0.828	0.755	0.813	0.828
C-17	0.817	0.874	0.834	0.787	0.786	0.820
C-18	0.816	0.882	0.826	0.789	0.794	0.825
C-19	0.819	0.873	0.762	0.772	0.798	0.810
C-20	0.825	0.869	0.840	0.787	0.796	0.832
E-1	0.847	0.883	0.845	0.797	0.861	0.829
E-2	0.848	0.883	0.891	0.785	0.859	0.855
E-5	0.847	0.879	0.876	0.797	0.867	0.831
E-10	0.838	0.879	0.836	0.784	0.871	0.857
E-20	0.844	0.877	0.864	0.785	0.850	0.849
I-60-40	0.837	0.874	0.853	0.810	0.851	0.836
I-67-33	0.846	0.876	0.852	0.802	0.861	0.832
I-75-25	0.847	0.877	0.831	0.799	0.853	0.831
I-80-20	0.842	0.873	0.862	0.803	0.868	0.830
I-40-30-30	0.848	0.879	0.805	0.805	0.858	0.861
I-60-20-20	0.844	0.879	0.817	0.800	0.857	0.846
I-80-10-10	0.845	0.872	0.866	0.784	0.865	0.819
I-60-30-10	0.853	0.877	0.828	0.795	0.855	0.854
I-30-30-15-15-10	0.848	0.881	0.833	0.803	0.845	0.826
I-60-10-10-10-10	0.841	0.874	0.828	0.786	0.858	0.821

Supplementary Table 8 Results for federated learning regression experiments utilizing benchmarked averaging strategy reported using the R^2 metric.

**Setting**	**CLint**	**FuBrain**	**FupHuman**	**FupRat**	**PappCaco2**	**Papp-LLC**	**RbRat**
Baseline	0.489	0.591	0.690	0.594	0.451	0.577	0.669
C-2	0.510	0.516	0.687	0.632	0.366	-0.502	0.616
C-3	0.379	0.492	0.684	0.619	0.349	0.572	0.522
C-4	0.477	0.398	0.665	0.621	0.350	-0.502	0.137
C-5	0.496	0.386	0.658	-	0.393	0.612	0.580
C-6	0.480	0.446	0.645	0.170	0.302	-0.227	0.210
C-7	0.442	0.351	0.673	0.640	0.270	0.583	0.346
C-8	0.462	0.358	0.651	0.433	0.285	0.561	0.354
C-9	0.484	0.362	-0.571	0.163	0.264	0.358	0.050
C-10	0.194	0.357	0.645	0.561	0.256	0.544	0.108
C-11	0.384	0.240	0.611	0.267	-0.216	-0.064	0.131
C-12	0.379	0.289	0.495	-0.012	-0.337	-0.502	0.111
C-13	0.402	0.271	-0.093	0.158	-0.206	-0.016	-0.030
C-14	0.413	0.347	0.018	0.228	-0.227	0.438	0.279
C-15	0.396	0.362	0.654	0.192	-0.209	0.420	0.528
C-16	0.370	0.388	0.630	0.600	-0.273	0.474	-0.118
C-17	0.351	0.328	0.624	-0.009	-0.277	0.556	0.441
C-18	0.345	0.270	0.616	0.220	-0.235	0.459	-0.048
C-19	0.312	0.280	0.600	0.414	-0.267	0.575	0.301
C-20	0.370	0.328	0.617	-0.190	-0.288	0.507	-0.014
E-1	0.515	0.453	0.115	0.639	0.401	0.486	-0.100
E-2	0.527	0.499	0.707	0.652	0.398	-	0.587
E-5	0.498	0.462	0.667	0.541	0.381	0.516	0.708
E-10	0.486	-	0.680	0.568	0.391	-0.471	0.396
E-20	0.493	-0.488	0.642	0.652	0.495	0.646	0.464
I-60-40	0.510	0.536	0.663	0.575	0.346	0.459	0.529
I-67-33	0.466	0.504	0.662	0.578	0.406	0.603	0.589
I-75-25	0.518	-0.016	0.672	0.558	-666.9	0.485	0.692
I-80-20	0.483	0.522	0.651	0.529	0.387	0.547	0.532
I-40-30-30	0.498	0.481	0.679	0.500	0.389	0.541	-0.109
I-60-20-20	0.520	0.496	0.651	-0.162	0.247	0.468	0.731
I-80-10-10	0.492	-0.370	-0.262	-0.361	-0.242	0.462	-0.588
I-60-30-10	0.494	0.463	0.632	0.655	0.406	-0.502	0.603
I-30-30-15-15-10	0.503	0.352	0.639	0.611	0.370	0.295	0.332
I-60-10-10-10-10	0.476	0.390	0.664	0.578	-0.125	0.268	-0.112

S3 Experiments using TDCommons datasets

kMoL is additionally evaluated on TDCommons [15] datasets, which include various Toxicity and ADME datasets. The results of the experiments are presented in Supplementary Table 9, while the optimal model hyperparameter values are presented in Supplementary Table 10.

Supplementary Table 9 Results for additional experiments using TDCommons [15] datasets. The *ROC-AUC* metric is reported for classification, and R^2 metric for regression tasks.

Dataset	ROC-AUC	R2
BBB (Blood-brain Barrier) [16]	0.987	-
BBB (Blood-brain Barrier) [17]	0.947	
Bioavailability [18]	0.787	
Caco-2 (Cell Effective Permeability) [19]	-	0.760
Carcinogens [20]	0.948	
Clearance [21]	-	0.028
Hepatocyte Clearance [22]	-	0.195
Microsome Clearance [22]	-	0.383
ClinTox [23]	0.951	-
CYP P450 1A2 Inhibition [24]	0.943	-
CYP P450 2C19 Inhibition [24]	0.903	-
CYP2C9 Substrate [25]	0.766	-
CYP P450 2C9 Inhibition [24]	0.900	-
CYP2D6 Substrate [25]	0.812	-
CYP P450 2D6 Inhibition [24]	0.893	-
CYP3A4 Substrate [25]	0.723	-
CYP P450 3A4 Inhibition [24]	0.915	-
DILI (Drug Induced Liver Injury) [26]	0.927	-
F20 [21]	0.782	-
F30 [21]	0.806	-
Half Life [21]	-	0.501
Half Life [27]	-	0.405
hERG [28]	0.914	-
HIA (Human Intestinal Absorption) [29]	0.998	-
Hydration Free Energy [30]	-	0.930
Acute Toxicity LD50 [31]	-	0.669
Lipophilicity [22]	-	0.763
P-gp (P-glycoprotein) Inhibition [32]	0.955	-
PPBR (Plasma Protein Binding Rate) [33]	-	0.641
PPBR (Plasma Protein Binding Rate) [21]	-	0.732
PPBR (Plasma Protein Binding Rate) [18]	0.885	-
Skin Reaction [34]	0.896	-
Solubility [35]	-	0.837
Volumn of Distribution [21]	-	0.121
Volumn of Distribution [36]	-	0.315

Supplementary Table 10 Optimal values for additional experiment model hyperparameters using TDCommons [15] datasets.

Dataset	Layer Type	Layers	Dropout	Optimizer	Residual
BBB (Blood-brain Barrier) [16]	SAGEConv [11]	7	0.2	AdamW [3]	Yes
BBB (Blood-brain Barrier) [17]	GENConv [13]	3	0.4	AdaBelief [4]	No
Bioavailability [18]	GCNConv [12]	2	0.3	AdaBelief [4]	No
Caco-2 (Cell Effective Permeability) [19]	GraphConv [8]	5	0.2	Adam [2]	No
Carcinogens [20]	GENConv[13]	7	0.0	AdamW [3]	Yes
Clearance [21]	GCNConv [12]	4	0.0	AdamW [3]	No
Hepatocyte Clearance [22]	GCNConv [12]	3	0.2	AdaBelief [4]	Yes
Microsome Clearance [22]	GraphConv [8]	7	0.2	Adam [2]	Yes
ClinTox [23]	GINConv [9]	7	0.2	AdaBelief [4]	No
CYP P450 1A2 Inhibition [24]	SAGEConv [11]	7	0.1	AdaBelief [4]	No
CYP P450 2C19 Inhibition [24]	GCNConv [12]	7	0.1	Adam [2]	No
CYP2C9 Substrate [25]	GraphConv [8]	7	0.4	Adam [2]	Yes
CYP P450 2C9 Inhibition [24]	GraphConv [8]	2	0.4	AdaBelief [4]	No
CYP2D6 Substrate [25]	SAGEConv [11]	5	0.0	AdaBelief [4]	No
CYP P450 2D6 Inhibition [24]	SAGEConv [11]	6	0.1	AdamW [3]	No
CYP3A4 Substrate [25]	LEConv [10]	4	0.0	SGD [1]	No
CYP P450 3A4 Inhibition [24]	GraphConv [8]	3	0.1	Adam [2]	Yes
DILI (Drug Induced Liver Injury) [26]	GCNConv [12]	6	0.4	SGD [1]	No
F20 [21]	GENConv [13]	3	0.2	AdamW [3]	Yes
F30 [21]	GraphConv [8]	6	0.4	Adam [2]	No
Half Life [21]	GINConv [9]	6	0.2	AdamW [3]	Yes
Half Life [27]	GCNConv [12]	3	0.2	AdamW [3]	No
hERG [28]	LEConv [10]	2	0.1	SGD [1]	Yes
HIA (Human Intestinal Absorption) [29]	GINConv [9]	4	0.0	AdamW [3]	Yes
Hydration Free Energy [30]	GENConv [13]	4	0.0	AdaBelief [4]	Yes
Acute Toxicity LD50 [31]	LEConv [10]	3	0.1	AdaBelief [4]	No
Lipophilicity [22]	GENConv [13]	5	0.1	AdamW [3]	No
P-gp (P-glycoprotein) Inhibition [32]	SAGEConv [11]	8	0.2	Adam [2]	Yes
PPBR (Plasma Protein Binding Rate) [33]	GraphConv [8]	4	0.1	AdamW [3]	No
PPBR (Plasma Protein Binding Rate) [21]	GCNConv [12]	3	0.1	Adam [2]	No
PPBR (Plasma Protein Binding Rate) [18]	GENConv [13]	7	0.2	SGD [1]	Yes
Skin Reaction [34]	GraphConv [8]	4	0.0	SGD [1]	Yes
Solubility [35]	SAGEConv [11]	4	0.0	Adam [2]	Yes
Volumn of Distribution [21]	GCNConv [12]	3	0.2	AdaBelief [4]	Yes
Volumn of Distribution [36]	GENConv [13]	2	0.3	Adam [2]	Yes

S4 Multimodal experiments using the ChEMBL dataset

A protein-ligand affinity dataset is created using a subset of the original ChEMBL [37] dataset, containing proteins with at least 100 positive or negative measurements. The protein information is stored as amino-acid sequences, and the ligand information is stored as SMILES strings. The pChEMBL value is used as an output feature and calculated as $-log_{10}(x)$, where x is the measured IC50, Ki, EC50, or Kd value.

The final version of the dataset consists of 947,586 entries containing 1,413 unique protein sequences and 583,624 unique ligands. Additionally, the experiments are conducted with three distinct classification threshold values alongside the raw regression values. The threshold values are 100 nM if the pChEMBL value is 7, 1 μM if the pChEMBL value is 6, and 10 μM if the pChEMBL value is 5, resulting in varying data distributions. The number of positive entries for each threshold value is 404,321, 645,186, and 852,925, with a corresponding number of negative entries of 543,265, 302,400, and 94,661, respectively.

The protein sequence encoding uses a bag-of-words featurization, while the ligand features are represented as graphs. The protein sequence features are processed through a multi-layer perceptron with 9,723 input features, 160 hidden features, and 16 output features. For the ligand features, a GENeralized Graph Network [13] with 5 layers, no dropout, no residual connections, and BatchNorm [5] for normalization was employed. The graph model has 192 hidden features.

SGD [1] is selected as the optimizer, with a learning rate of 0.0041 and a weight decay rate of 0.00001. Binary cross-entropy loss is utilized for classification, while Smooth L1 loss is utilized for regression tasks. A batch size of 128 is used, and the models are trained for a total of 50 epochs. For the regression task, a log transformation is applied to the target values.

Supplementary Table 11 Results for multimodal experiments using the ChEMBL [37] dataset.

Threshold Value	Metric	Metric Value
-	R^2	0.651
100 nM	$ROC\text{-}AUC$	0.891
1 μm	$ROC\text{-}AUC$	0.900
10 μm	$ROC\text{-}AUC$	0.916

References

[1] Kiefer, J., Wolfowitz, J.: Stochastic estimation of the maximum of a regression function. The Annals of Mathematical Statistics **23**(3), 462–466 (1952). Full publication date: Sep., 1952

[2] Kingma, D.P., Ba, J.: Adam: A Method for Stochastic Optimization (2017)

[3] Loshchilov, I., Hutter, F.: Decoupled Weight Decay Regularization (2019)

[4] Zhuang, J., Tang, T., Ding, Y., Tatikonda, S., Dvornek, N., Papademetris, X., Duncan, J.S.: AdaBelief Optimizer: Adapting Stepsizes by the Belief in Observed Gradients (2020)

[5] Ioffe, S., Szegedy, C.: Batch Normalization: Accelerating Deep Network Training by Reducing Internal Covariate Shift (2015)

[6] Ulyanov, D., Vedaldi, A., Lempitsky, V.: Instance Normalization: The Missing Ingredient for Fast Stylization (2017)

[7] Cai, T., Luo, S., Xu, K., He, D., Liu, T.-Y., Wang, L.: GraphNorm: A Principled Approach to Accelerating Graph Neural Network Training (2021)

[8] Morris, C., Ritzert, M., Fey, M., Hamilton, W.L., Lenssen, J.E., Rattan, G., Grohe, M.: Weisfeiler and Leman Go Neural: Higher-order Graph Neural Networks (2020)

[9] Xu, K., Hu, W., Leskovec, J., Jegelka, S.: How Powerful are Graph Neural Networks? (2019)

[10] Ranjan, E., Sanyal, S., Talukdar, P.P.: ASAP: Adaptive Structure Aware Pooling for Learning Hierarchical Graph Representations (2020)

[11] Hamilton, W.L., Ying, R., Leskovec, J.: Inductive Representation Learning on Large Graphs (2018)

[12] Thomas N. Kipf, M.W.: Semi-supervised classification with graph convolutional networks. Arxiv (2017)

[13] Li, G., Xiong, C., Thabet, A., Ghanem, B.: DeeperGCN: All You Need to Train Deeper GCNs (2020)

[14] Li, P., Li, Y., Hsieh, C.-Y., Zhang, S., Liu, X., Liu, H., Song, S., Yao, X.: TrimNet: learning molecular representation from triplet messages for biomedicine. Briefings in Bioinformatics (2020) https://doi.org/10.1093/bib/bbaa266 https://academic.oup.com/bib/advance-article-pdf/doi/10.1093/bib/bbaa266/34130210/bbaa266.pdf. bbaa266

[15] Huang, K., Fu, T., Gao, W., Zhao, Y., Roohani, Y., Leskovec, J., Coley, C.W., Xiao, C., Sun, J., Zitnik, M.: Therapeutics data commons: Machine learning datasets and tasks for drug discovery and development. Proceedings of Neural Information Processing Systems, NeurIPS Datasets and Benchmarks (2021)

[16] Adenot, M., Lahana, R.: Blood-brain barrier permeation models: Discriminating between potential cns and non-cns drugs including p-glycoprotein substrates. Journal of Chemical Information and Computer Sciences **44**(1), 239–248 (2004) https://doi.org/10.1021/ci034205d https://doi.org/10.1021/ci034205d. PMID: 14741033

[17] Martins, I.F., Teixeira, A.L., Pinheiro, L., Falcao, A.O.: A bayesian approach to in silico blood-brain barrier penetration modeling. Journal of Chemical Information and Modeling **52**(6), 1686–1697 (2012) https://doi.org/10.1021/ci300124c https://doi.org/10.1021/ci300124c. PMID: 22612593

[18] Ma, C.-Y., Yang, S.-Y., Zhang, H., Xiang, M.-L., Huang, Q., Wei, Y.-Q.: Prediction models of human plasma protein binding rate and oral bioavailability derived by using ga–cg–svm method. Journal of Pharmaceutical and Biomedical Analysis **47**(4), 677–682 (2008) https://doi.org/10.1016/j.jpba.2008.03.023

[19] Wang, N.-N., Dong, J., Deng, Y.-H., Zhu, M.-F., Wen, M., Yao, Z.-J., Lu, A.-P., Wang, J.-B., Cao, D.-S.: Adme properties evaluation in drug discovery: Prediction of caco-2 cell permeability using a combination of nsga-ii and boosting. Journal of Chemical Information and Modeling **56**(4), 763–773 (2016) https://doi.org/10.1021/acs.jcim.5b00642 https://doi.org/10.1021/acs.jcim.5b00642. PMID: 27018227

[20] Lagunin, A., Filimonov, D., Zakharov, A., Xie, W., Huang, Y., Zhu, F., Shen, T., Yao, J., Poroikov, V.: Computer-aided prediction of rodent carcinogenicity by pass and cisoc-psct. QSAR & Combinatorial Science **28**(8), 806–810 (2009) https://doi.org/10.1002/qsar.200860192 https://onlinelibrary.wiley.com/doi/pdf/10.1002/qsar.200860192

[21] Pihan, E., Colliandre, L., Guichou, J.-F., Douguet, D.: e-drug3d: 3d structure collections dedicated to drug repurposing and fragment-based drug design. Bioinformatics **28**(11), 1540–1541 (2012) https://doi.org/10.1093/bioinformatics/bts186 https://academic.oup.com/bioinformatics/article-pdf/28/11/1540/48868517/bioinformatics_28_11_1540.pdf

[22] AstraZeneca: Experimental in vitro Dmpk and physicochemical data on a set of publicly disclosed compounds (2016). https://doi.org/10.6019/chembl3301361

[23] Gayvert, K.M., Madhukar, N.S., Elemento, O.: A data-driven approach to predicting successes and failures of clinical trials. Cell Chemical Biology **23**(10), 1294–1301 (2016) https://doi.org/10.1016/j.chembiol.2016.07.023

[24] DS;, V.H.N.R.T.D.N.M.J.C.D.: Comprehensive characterization of cytochrome P450 isozyme selectivity across chemical libraries. U.S. National Library of Medicine. https://pubmed.ncbi.nlm.nih.gov/19855396/

[25] Carbon-Mangels, M., Hutter, M.C.: Selecting relevant descriptors for classification by bayesian estimates: A comparison with decision trees and support vector machines approaches for disparate data sets. Molecular Informatics **30**(10), 885–895 (2011) https://doi.org/10.1002/minf.201100069 https://onlinelibrary.wiley.com/doi/pdf/10.1002/minf.201100069

[26] Xu, Y., Dai, Z., Chen, F., Gao, S., Pei, J., Lai, L.: Deep learning for drug-induced liver injury. Journal of Chemical Information and Modeling **55**(10), 2085–2093 (2015) https://doi.org/10.1021/acs.jcim.5b00238 https://doi.org/10.1021/acs.jcim.5b00238. PMID: 26437739

[27] Obach, R.S., Lombardo, F., Waters, N.J.: Trend analysis of a database of intravenous pharmacokinetic parameters in humans for 670 drug compounds. Drug Metabolism and Disposition **36**(7), 1385–1405 (2008) https://doi.org/10.1124/dmd.108.020479 https://dmd.aspetjournals.org/content/36/7/1385.full.pdf

[28] Wang, S., Sun, H., Liu, H., Li, D., Li, Y., Hou, T.: Admet evaluation in drug discovery. 16. predicting herg blockers by combining multiple pharmacophores and machine learning approaches. Molecular Pharmaceutics **13**(8), 2855–2866 (2016) https://doi.org/10.1021/acs.molpharmaceut.6b00471 https://doi.org/10.1021/acs.molpharmaceut.6b00471. PMID: 27379394

[29] Hou, T., Wang, J., Zhang, W., Xu, X.: Adme evaluation in drug discovery. 7. prediction of oral absorption by correlation and classification. Journal of Chemical Information and Modeling **47**(1), 208–218 (2007) https://doi.org/10.1021/ci600343x https://doi.org/10.1021/ci600343x. PMID: 17238266

[30] Mobley David L., G.J.P.: FreeSolv: a database of experimental and calculated hydration free energies, with input files. Journal of Computer-Aided Molecular Design. https://doi.org/10.1007/s10822-014-9747-x

[31] Zhu, H., Martin, T.M., Ye, L., Sedykh, A., Young, D.M., Tropsha, A.: Quantitative structure-activity relationship modeling of rat acute toxicity by oral exposure. Chemical Research in Toxicology **22**(12), 1913–1921 (2009) https://doi.org/10.1021/tx900189p https://doi.org/10.1021/tx900189p. PMID: 19845371

[32] Broccatelli, F., Carosati, E., Neri, A., Frosini, M., Goracci, L., Oprea, T.I., Cruciani, G.: A novel approach for predicting p-glycoprotein (abcb1) inhibition using molecular interaction fields. Journal of Medicinal Chemistry **54**(6), 1740–1751 (2011) https://doi.org/10.1021/jm101421d https://doi.org/10.1021/jm101421d. PMID: 21341745

[33] Wenlock, M., Tomkinson, N.: Experimental in vitro DMPK and physicochemical data on a set of publicly disclosed compounds. http://dx.doi.org/10.6019/CHEMBL3301361

[34] Alves, V.M., Muratov, E., Fourches, D., Strickland, J., Kleinstreuer, N., Andrade, C.H., Tropsha, A.: Predicting chemically-induced skin reactions. part i: Qsar models of skin sensitization and their application to identify potentially hazardous compounds. Toxicology and Applied Pharmacology **284**(2), 262–272 (2015) https://doi.org/10.1016/j.taap.2014.12.014

[35] Murat Cihan Sorkun, A.K..S.E.: AqSolDB, a curated reference set of aqueous solubility and 2D descriptors for a diverse set of compounds. Nature Scientific Data. https://www.nature.com/articles/s41597-019-0151-1

[36] Lombardo, F., Jing, Y.: In silico prediction of volume of distribution in humans. extensive data set and the exploration of linear and nonlinear methods coupled with molecular interaction fields descriptors. Journal of Chemical Information and Modeling **56**(10), 2042–2052 (2016) https://doi.org/10.1021/acs.jcim.6b00044 https://doi.org/10.1021/acs.jcim.6b00044. PMID: 27602694

[37] Gaulton, A., Hersey, A., Nowotka, M., Bento, A.P., Chambers, J., Mendez, D., Mutowo, P., Atkinson, F., Bellis, L.J., Cibrián-Uhalte, E., Davies, M., Dedman, N., Karlsson, A., Magariños, M.P., Overington, J.P., Papadatos, G., Smit, I., Leach, A.R.: The chembl database in 2017. Nucleic Acids Research **45**(D1), 945–954 (2016) https://doi.org/10.1093/nar/gkw1074 https://academic.oup.com/nar/article-pdf/45/D1/D945/8846762/gkw1074.pdf
